# The conquering of North America: dated phylogenetic and biogeographic inference of migratory behavior in bee hummingbirds

**DOI:** 10.1186/s12862-017-0980-5

**Published:** 2017-06-05

**Authors:** Yuyini Licona-Vera, Juan Francisco Ornelas

**Affiliations:** Departamento de Biología Evolutiva, Instituto de Ecología, A.C., Carretera Antigua a Coatepec No. 351, El Haya, Xalapa, 91070 Veracruz, Mexico

**Keywords:** Bee hummingbirds, Biogeography, Mellisugini, Molecular phylogeny, Migration, North America

## Abstract

**Background:**

Geographical and temporal patterns of diversification in bee hummingbirds (Mellisugini) were assessed with respect to the evolution of migration, critical for colonization of North America. We generated a dated multilocus phylogeny of the Mellisugini based on a dense sampling using Bayesian inference, maximum-likelihood and maximum parsimony methods, and reconstructed the ancestral states of distributional areas in a Bayesian framework and migratory behavior using maximum parsimony, maximum-likelihood and re-rooting methods.

**Results:**

All phylogenetic analyses confirmed monophyly of the Mellisugini and the inclusion of *Atthis*, *Calothorax*, *Doricha*, *Eulidia*, *Mellisuga*, *Microstilbon*, *Myrmia*, *Tilmatura*, and *Thaumastura*. Mellisugini consists of two clades: (1) South American species (including *Tilmatura dupontii*), and (2) species distributed in North and Central America and the Caribbean islands. The second clade consists of four subclades: Mexican (*Calothorax*, *Doricha*) and Caribbean (*Archilochus*, *Calliphlox*, *Mellisuga*) sheartails, *Calypte*, and *Selasphorus* (incl. *Atthis*). Coalescent-based dating places the origin of the Mellisugini in the mid-to-late Miocene, with crown ages of most subclades in the early Pliocene, and subsequent species splits in the Pleistocene. Bee hummingbirds reached western North America by the end of the Miocene and the ancestral mellisuginid (bee hummingbirds) was reconstructed as sedentary, with four independent gains of migratory behavior during the evolution of the Mellisugini.

**Conclusions:**

Early colonization of North America and subsequent evolution of migration best explained biogeographic and diversification patterns within the Mellisugini. The repeated evolution of long-distance migration by different lineages was critical for the colonization of North America, contributing to the radiation of bee hummingbirds. Comparative phylogeography is needed to test whether the repeated evolution of migration resulted from northward expansion of southern sedentary populations.

**Electronic supplementary material:**

The online version of this article (doi:10.1186/s12862-017-0980-5) contains supplementary material, which is available to authorized users.

## Background

Bird migration is one of the most extraordinary behaviors found in nature. The voyage for migration involves a fascinating suite of characters including navigational systems, physiological specializations and the seasonal timing of events [1, 2]. Our knowledge of several ecological aspects of migration has become impressive over time [3, 4]; however, much remains to be learned on how long-distance seasonal migration repeatedly evolved in a wide variety of bird lineages and about the selection pressures underlying the evolution of migration [5–8]. In particular, the origin and geographical directionality of long-distance seasonal migration has been widely debated in the literature (e.g., [7]), centered in two prominent ideas originated on the examination of current distributions of migratory species and their presumed sister species: the ‘southern-home’ and the ‘northern-home’ hypotheses. The ‘southern-home’ hypothesis posits that the breeding migratory species from the temperate regions are returning to their tropical ancestral ranges during the winter, whereas the ‘northern-home’ hypothesis postulates that the ancestral temperate range of migratory species becomes harsh for survival and depart to the novel tropics during the winter, and then returning to their ancestral home for breeding [6, 9]. In the ‘southern-home’ hypothesis, it is assumed that migration should evolve from sedentary ancestors to migratory descendants in response to ecological change or vice versa in the ‘northern-home’ hypothesis [10].

Escaping from intraspecific competition and the environmental seasonality with low food availability during the breeding season has been interpreted as being crucial for the evolution of migration [6, 11–13]. However, other factors such as increased harshness of climatic conditions and variation in resource availability during the non-breeding season, predation or parasitism would also make species to shift their breeding ranges and become migrants [6, 12]. Likely, migrant populations originating from southern tropical regions might have shifted their ranges northwards through long-distance dispersal coupled with climatic cycles [14], assuming competition in the tropical breeding ranges or the use of seasonally abundant resources in temperate regions as the driving forces for the northward expansion and evolution of migration [2, 6, 9, 15]. Several authors have envisioned scenarios for the transition from a sedentary to a migratory species over evolutionary time [9, 11, 12, 16]. As a result of the differential effects of intraspecific and interspecific competition, increasing seasonality of climate or by certain patterns of climatic change during the Tertiary and Pleistocene glaciations, Cox [11] proposed that migration evolves from changing the initial sedentary condition to that of a partial migrant, having with both permanent sedentary populations and populations migrating into seasonally favorable adjacent areas. Partial migrants then evolve further through extinction of sedentary populations and expansion into derived forms with separate or disjunct seasonal ranges. In contrast, Levey and Stiles [16] developed a scenario where temporal and spatial variation of resources, especially for fruit- and nectar-feeding birds, led to altitudinal intra-tropical migration, predisposing these birds to migrate out of the tropics.

Despite the appeal of intraspecific competition and variation in resource availability as being the first step for the evolution of migration, these scenarios have several shortcomings (reviewed in [6]) including those that have shown how in a small fraction of recently-expanded populations migratory behavior can increase rapidly when favored by selection (e.g., [17, 18]). Therefore, the repeated evolution of long-distance seasonal migration within bird lineages linked to the occurrence of relatively fast range expansions to take advantage of abundant resources could be the result of selective pressures occurring throughout several climatic cycles affecting resource availability in seasonally changing environments.

More recently, Somveille and collaborators [19–22] examined global spatial patterns in the diversity of migratory birds, and found strong support for the hypothesis that seasonality is the main force driving bird migration worldwide (see also [23]). Whereas the previous studies attempt to explain the ecological mechanisms driving the higher diversity of migratory species in the Northern Hemisphere [22, 23], Rolland et al. [8] used molecular phylogenies that included most extant bird species to infer that sedentary behavior is ancestral and that migratory behavior evolved independently multiple times during the evolutionary history of birds. They also found that seasonal migration increases diversification via sedentary populations arising from migratory populations (asymmetrical speciation), in which speciation of ancestral species into one sedentary and one migratory species was more frequent in migratory species than sedentary. Their results suggest that the evolution of seasonal migration in birds has facilitated diversification through the divergence of migratory subpopulations that become sedentary, and illustrate asymmetrical diversification as a mechanism by which diversification rates are decoupled from species richness.

Hummingbirds (Trochilidae) are one of the largest bird families, with ca. 338 species distributed in the Americas [24]. The most recent molecular phylogeny suggests that hummingbirds split from their sister group, swifts and treeswifts, ca. 42 million years ago (MYA) in Eurasia and that the age of the common ancestor of hummingbirds in South America is ca. 22 MYA [25]. Given the gap between these two events and the absence of relevant fossils in the Americas, McGuire et al. [25] hypothesized that hummingbirds reached North America by dispersal across Beringia. After that, hummingbirds dispersed to the South American continent and may have become extinct both in Europe and North America [25]. Hummingbirds have diversified into nine clades (Topazes, Hermits, Mangoes, Brilliants, Coquettes, *Patagona*, Mountain Gems, Bees, Emeralds), seven of which rapidly diversified in South America in conjunction with the Andean uplift. The common ancestor of the other two clades, Bees and Mountain Gems, recolonized North America ca. 12 MYA [25], before the formation of the Central American land bridge and closure of the Isthmus of Panama. While hummingbird diversification probably increased in conjunction with the Andean uplift according to divergence dating using substitution rate priors (rather than fossil calibrations) [25], other divergence-dating analyses using both fossil calibrations and substitution rate priors retrieved older divergence splits between Bees and Mountain Gems (20–25 MYA; [26]), suggesting that North American hummingbirds are not recent colonizers and may have only become extinct in Europe [26, 27].

The ‘bee’ hummingbirds (Mellisugini tribe; [24]) comprise an assemblage of 16 genera and 36 small species distributed throughout the Americas, from southern Canada to South America [28]. Although some species are geographically widespread (e.g., *Archilochus* spp.; [28]), other have very restricted distributions such as the smallest bird of the world (*Mellisuga helenae*) endemic to Cuba. The most extensive molecular phylogeny of hummingbirds to date [25], with at least one representative species for each genus in the Mellisugini, estimated its relatively recent origin (ca. 5 MYA), revealed a high rate of diversification (0.57 species/MYA), as compared to other hummingbird clades. This phylogeny retrieved Mellisugini as composed of two main clades: one clade included species informally named “woodstars” distributed in South America and *Tilmatura dupontii* with distribution in Central and North America, and the second clade contained species arranged as in two subclades: (1) *Calypte*, *Selasphorus* and *Atthis* species, and (2) “sheartails” (*Doricha eliza* and *Calothorax lucifer*), *Archilochus* (*A. colubris* and *A. alexandri*), *Calliphlox evelynae* and *Mellisuga minima*, in which phylogenetic relationships between the “sheartails” and the other species within the subclade are not supported. Besides the high rate of diversification, Mellisugini species are distinguished by the dimorphic tail morphology, which in males the rectrices are unusual in shape to produce sounds and acrobatic courtship displays during the breeding season (e.g., [29–32]).

Mellisugini is the only group of hummingbirds with long-distance seasonal migration and, therefore, an interesting study group from a biogeographic perspective. Most of the species in Canada and the USA are obligate, long-distance seasonal migrants, which vacate their entire breeding range to winter mainly in Mexico [28]. Several aspects of hummingbird long-distance seasonal migration are particularly remarkable, with journeys across the Gulf of Mexico by *Archilochus colubris* or those of more than 6000 km by *Selasphorus rufus*, breeding in western United States and Canada and overwintering in Mexico [33]. However, the origin and evolution of migratory behavior and the impact on hummingbird diversification has not been studied. The evolution of hummingbird migration is a complex phenomenon to address because it is thought to evolve rapidly in response to selection [9, 34–36]. Previous phylogenetic hypothesis [25] suggests that migratory behavior is not evolutionarily constrained, as both sedentary and long-distance migratory species seem to have evolved repeatedly within the Mellisugini. Understanding the evolution of migratory behavior within the Mellisugini is important, particularly because they are susceptible to rapid evolutionary change, i.e. their high rate of net diversification with species accumulation during their brief 5 MYA history [25], and because they can change their migratory behavior to escape from increased harshness of climatic conditions during the Pleistocene glacial cycles [37], and from seasonal changes in the phenology and availability of nectar floral resources by current global climate changes [38]. Unfortunately, the lack of a wider geographic sampling and the absence of some North American representative species from previous phylogenetic analyses, has not allowed having a fully resolved phylogeny of the group to understanding the evolution of long-distance seasonal migration and timing of diversification and colonization patterns.

The objectives of our study were to: (1) reconstruct the phylogenetic relationships among bee hummingbirds increasing both geographical and intraspecific sampling, (2) estimate divergence times between species and genera, and (3) reconstruct the ancestral range at each divergence event, and subsequent temporal and geographical shifts on migration in bee hummingbirds. The suite of morphological and behavioral characters coupled with the wide variety of environments where they live, including the most xeric environments tolerated within hummingbirds, have been linked to their relatively rapid radiation with highest rate of species’ accumulation [25]. Thus, the Mellisugini present a useful model for exploring hidden biodiversity due to its wide distribution in both North and South American continents and recent biogeographic origin, and for understanding the potential impact of shifts between sedentary and long-distance migratory behavior on diversification of bee hummingbirds because migratory and non-migratory species, and species with partial migration (migratory and non-migratory populations) occur only in the North American continent.

## Methods

### Sampling and laboratory methods

The data set included 116 samples of bee hummingbirds from North America and the Caribbean Islands and 1–2 samples of bee hummingbirds from South America (*n* = 16 samples), representing all 16 genera of bee hummingbirds (32 of the 36 extant species, 89%). Tissue samples were unavailable for four species: *Chaetocercus astreans* (Colombia), *C. berlepschi* (Ecuador), *C. heliodor* (Colombia, Venezuela and Ecuador), and *Mellisuga helenae* (Cuba). Most of these species are endemic and range-restricted; *Chaetocercus berlepschi* is threatened by habitat loss [39, 40]. We include new sequence data for 60 individuals from the genera *Archilochus*, *Atthis*, *Calothorax*, *Doricha*, *Calypte*, *Selasphorus* and *Tilmatura* to supplement the data set in McGuire et al. [25] and Feo et al. [31]. Additionally, we included a single individual of each of 15 species of mountain gems and emeralds to be used for sequence alignment and as outgroups. Samples were obtained from vouchered tissue collections (see Acknowledgements) and from our collecting efforts in Mexico.

DNA was extracted from tissue or tail feathers with the DNeasy Tissue extraction kit (Qiagen, Valencia, CA, USA) using the standard protocol. We amplified and sequenced six gene regions, two mitochondrial protein coding genes—1041 base pairs (bp) of nicotinamide dehydrogenase subunit 2 (*ND2*) and 807 bp of nicotinamide dehydrogenase subunit 4 (*ND4*), and four nuclear loci—1085 bp of fibrinogen beta chain intron (*FBG I7*), 551 bp of adenylate kinase 1 intron 5 (*AK1 I5*), 577 bp of ornithine decarboxylase 1 introns 6 and 7 intervening exon (*ODC1*), and 635 bp of Z-linked muscle, skeletal, receptor tyrosine kinase intron 3 (*MUSK I3*) using specific primers (Additional file 1). Protocols for PCR reactions and for sequencing the PCR products are described elsewhere [41]. The products were read on a 310 automated DNA sequencer (Applied Biosystems) at the INECOL’s sequencing facility. Finally, assembled sequences were edited and checked for quality, pre-aligned using MAFFT v7 (http://mafft.cbrc.jp/alignment/server/), and then manually aligned using PhyDE [42]. Newly generated sequences have been submitted to GenBank (Accession nos. *ND2*: KX855335– KX855393; *ND4*: KX855394– KX855450; *AK1 I5*: KX855451– KX855509; *MUSK I3*: KX855568– KX855624; *ODC1*: KX855510– KX855567; *FBG I7*: KX855625– KX855637; Additional file 2). The alignments supporting the results of this article are available in the Dryad Digital Repository (10.0000/dryad.t00h0) as Licona-Vera and Ornelas [43].

### Phylogenetic reconstruction

The phylogeny was reconstructed using Bayesian inference (BI), maximum-likelihood (ML) and maximum parsimony (MP). We performed BI comparative phylogenetic analyses using MrBAYES v3.2.2 [44] and the CIPRES Science Gateway [45] on the following data sets: (1) only mitochondrial genes (‘unpartitioned mtDNA’), (2) only mitochondrial genes as two partitions (‘partitioned mtDNA’), (3) only nuclear genes (‘unpartitioned nuDNA’), (4) only nuclear genes as four partitions (‘partitioned nuDNA’), (5) combined loci data set with a single model (‘concatenated’), (6) each DNA region as one partition (‘mtDNA + nuDNA’), and (7) with a set partition-specific DNA evolution models of each gene (‘6-partitions’). We used jMODELTEST v2.1.7 [46] to select an appropriate model of nucleotide substitution for each locus and the concatenated data set. GTR + I + G (*ND2*), TrN + I + G (*ND4*), K80 + G (*AK1 I5*), HKY (*MUSK I3*), HKY (*ODC1*), HKY + G (*FBG I7*), GTR + I + G (mtDNA data set), GTR + G (nuDNA data set), and TrN + I + G (concatenated) were selected as the best fitting models and incorporated as prior information in the Bayesian analyses. For each data set, two parallel Markov chain Monte Carlo (MCMC) analyses were executed simultaneously for 30 million generations, sampling every 10,000 generations. Output parameters were visualized using TRACER v1.6 (http://tree.bio.ed.ac.uk/software/tracer/). A 25% burn-in was used, and a majority rule consensus tree was calculated and visualized in FIGTREE v1.4.3 (http://tree.bio.ed.ac.uk/software/figtree/). We computed Bayes factors with the harmonic means [47] to determine whether applying partition-specific models for the combined data sets significantly improved the explanation of the data.

The ML analysis for the concatenated data set was run using RAxML v8.2.9 [48] with a GTRGAMMA model for each partition. Node support for the ML tree was estimated with 1000 bootstrap replicates.

The MP analysis was run for the concatenated data set in NONA v2.0 [49] using WINCLADA [50], with nucleotide characters treated as equally weighted and unordered. We ran 1000 iterations, holding 10 trees per iteration with 10%of the nodes constrained, and all the parameters set to default. Branch support was assessed using bootstrap resampling, 1000 bootstrap-resampled pseudo-replicate matrices were each analyzed using 100 random addition sequences (multi*100). Ten trees were retained during TBR swapping after each search initiation (hold/10).

### Divergence time estimation

A Bayesian relaxed-clock analysis was performed in BEAST v2.4.4 [51, 52] to assess species divergence times using the six genes. We constrained Trochilidae and the hummingbird clades used as outgroups (emeralds and mountain gems) as monophyletic based on McGuire et al. [25]. Divergence times were estimated using an uncorrelated lognormal relaxed clock model across all genes, with the trees linked and the substitution models for each partition unlinked [53]. We calibrated our divergence-dating analyses using a Yule speciation model and three calibration strategies for divergence time estimation: (1) incorporating a separate normally distributed substitution rate calibration priors for *ND2*, *ND4, AK1*, *FGB*, and *ODC* using the mean substitution rates proposed by Lerner et al. [54] to model the tree prior, allowing the substitution rate prior for *MUSK I3* to be calculated by BEAST because no substitution rate was available; (2) using as secondary calibration the age of the split between mountain gems and bee hummingbirds (normal, mean 12.0 MYA, SD ± 1, range of 13.9–10.3 MYA) according to McGuire et al. [25] to calibrate the root of the tree; and (3) using both strategies, secondary calibration + substitution rates. This strategy was also used for divergence time estimation using a reduced data set, which includes one individual for each of the species to contrast results of divergence time estimation from single vs. multiple-individuals data sets.

Two independent chains of MCMC were run with 50 million generations, sampling every 5000 generations. Results were visualized in TRACER v1.6 (http://tree.bio.ed.ac.uk/software/tracer/) to confirm appropriate burn-in, adequate effective samples sizes (ESS > 200) of the posterior distribution for all parameters, and to assess convergence among runs by comparing likelihoods of parameters. The three independent runs were combined with LOGCOMBINER v2.4.4 [51, 52] and the resulting maximum clade credibility tree and 95% highest posterior (HPD) distributions of each estimated node annotated using TREEANNOTATOR v2.4.4 [51, 52] and visualized in FIGTREE v1.4.3 (http://tree.bio.ed.ac.uk/).

### Ancestral areas of bee hummingbirds

We reconstructed ancestral geographic ranges using Bayesian methods with BBM (Bayesian Binary MCMC) analyses implemented in RASP v3.2.1 [55]. This method determines the probability of each ancestral geographical region for each node averaged over the collection of trees derived from a Bayesian MCMC analysis [56, 57]. To reconstruct the ancestral areas, we loaded 6000 trees from the Bayesian Inference analyses using MrBAYES. The breeding distributions of each sample was obtained from del Hoyo et al. [28] and crossed with the status and distribution information compiled by the Cornell Laboratory of Ornithology as input (www.allaboutbirds.org/guide). We coded each individual in the data set as occurring in one or more of the following areas: A = western North America, B = eastern North America, C = southeastern Mexico and Central America, D = West Indies, and E = South America (Additional file 3). These regions were based on a modified map of the ecoregions (http://maps.tnc.org/gis_data.html) proposed by Blair and Sánchez-Ramírez [58]. The posterior probabilities for nodes in the phylogeny with >0.90 were estimated to incorporate information from most nodes of the tree but minimizing phylogenetic ‘noise’ from poorly supported relationships. The maximum number of areas in ancestral ranges was constrained to three, *Amazilia rutila* assigned as outgroup using the ‘custom’ option, and the ancestral areas for nodes visualized on the condensed tree. Analyses were run for 50,000 iterations, sampling every 100 generations, the first 25% of which were discarded as burn-in, with the JC + gamma model of state transitions used as input.

### Evolution of migratory behavior

Ancestral state reconstruction was used to map migratory behavior onto the resulting molecular phylogeny. The evolution of migratory behavior was reconstructed using maximum parsimony (MP) and maximum-likelihood (ML) methods. We traced the evolution of migratory behavior over the molecular phylogeny using two topologies: one with all samples and the other with one sample per species. The first topology corresponds to the best estimate of Mellisugini phylogenetic relationships using the 6-partitions data set (see Results), a Bayesian 50% majority rule consensus tree of 132 samples of bee hummingbirds. We used the 18,000 post-burnin trees from the BEAST analysis to account for the phylogenetic uncertainty in the ancestral state reconstruction. The second topology was a Bayesian 50% majority rule consensus tree using one sample per species (see Results). This tree was obtained from a BEAST analysis using the same parameters and the 6-partitions strategy described on methods section of phylogenetic reconstruction.

For ancestral state reconstruction we used three different coding schemes mainly based on information in del Hoyo et al. [28] and Malpica and Ornelas ([37]; Additional files 3 and 4). In coding Scheme 1 species that migrate seasonally between different latitudinal geographical breeding and wintering ranges were coded as migrants (i.e., obligate, long-distance migration; [59]) and non-migratory species were coded as sedentary. For this coding scheme, we also considered as migratory those species with partial migration, in which some individuals or populations are fully migratory across their range and other individuals or populations are sedentary (*Selasphorus platycercus* and *Calothorax lucifer*; [60]). The migratory state does not include tropical hummingbird species that may undertake altitudinal or short-distance migration at the fringes of their northern ranges in the northern temperate region (e.g., *Amazilia violiceps*, *Eugenes fulgens*, *Heliomaster constantii*; [59]). In coding Schemes 2 and 3, species with partial migration were coded as sedentary or polymorphic, respectively, to test the robustness of our conclusions to potential ambiguities in character state coding [60]. Coding species with a simple binary codification (sedentary or migrant) and pruning trees to species probably mask or confuse the ancestral character reconstruction of species susceptible to rapid evolutionary change of migratory behavior [34–36]. Thus, we conducted ancestral state reconstruction using the data set with multiple individuals for a given species to compare results with those obtained for species-level analyses (single-individual data set). Also, insights might be gained from sampling several individuals if these provide the signal at the phylogenetic level of when the shifts from migratory to sedentary (or vice versa) occurred between populations in species with both sedentary and migratory populations. Here, single individuals of *C. lucifer* and *S. platycercus* with migratory and sedentary populations were classified as either migrant or sedentary based on data on Malpica and Ornelas [37] and because samples of single individuals were collected during the breeding season from known allopatric migratory or sedentary populations. Species names, English names and distributional range for the Mellisugini species used in this study are provided in Additional file 5.

MP and ML based ancestral state reconstruction were conducted in Mesquite v3.11 [61] using each of the coding schemes of migratory behavior described above. To account for topological uncertainty we used the ‘trace character over trees’ option, which summarizes the ancestral state reconstruction over a series of trees. All reconstructions were integrated over the last 18,000 post burn-in of the Bayesian analysis and the ancestral states were summarized using the ‘Count trees with uniquely best states’ option on the maximum credibility tree using Mesquite. A reconstruction is regarded as equivocal when there are two or more equally parsimonious states inferred at a particular node. For the parsimony ancestral character reconstructions character-state changes were set as unordered, with other parameters as default. In the ML approach, the character state for each ancestral node was reconstructed using the Markov k-state 1 parameter model (Mk1), which specifies an equal probability of any state change and considers the rate of change the only parameters. We conducted ancestral state reconstruction with more than one method because each of the two methods described above suffers from certain advantages and limitations [62–66].

Ancestral states of migratory behavior in the Mellisugini were also reconstructed using the re-rooting method of Yang et al. [67] as implemented in PHYTOOLS v0.5 [68] in R v3.3.0 [69]. This method re-roots the phylogeny at every node and calculates the phylogenetically independent contrast for the root node, taking advantage of the fact that this value is the maximum likelihood estimate for that node [70, 71]. We used the Mk1 model for reconstruction of the character state for each ancestral node, assuming equal rate of evolution. Since the likelihood approach is not applicable for polymorphic characters this reconstruction was performed using only the parsimony approach.

## Results

### Phylogeny

**Fig. 1:**
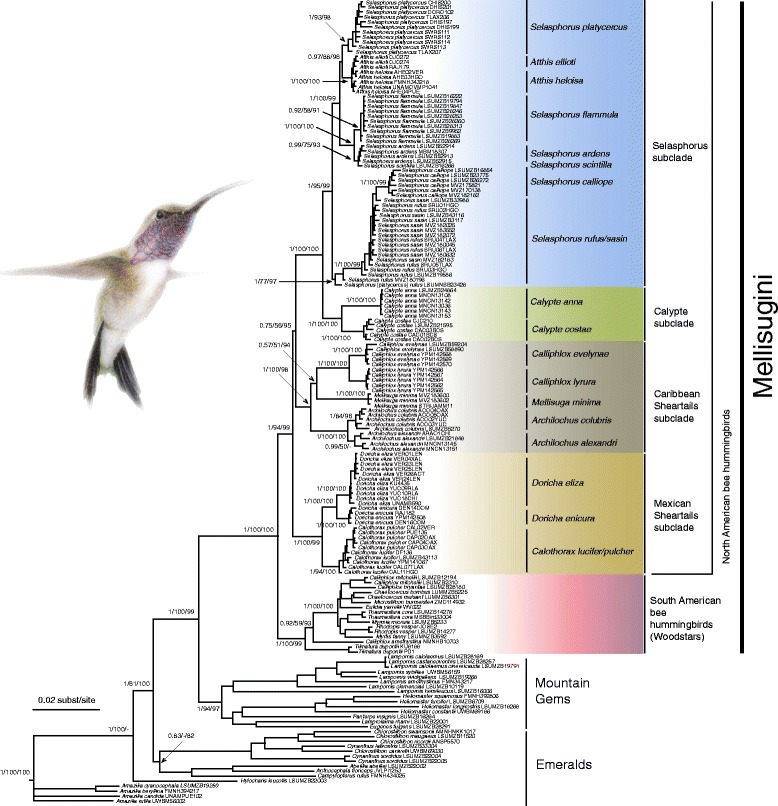
Phylogenetic 50% majority-rule consensus tree of the Mellisugini hummingbird from the Bayesian analysis of the combined NADH dehydrogenase subunit 2 (*ND2*) and subunit 4 (*ND4*) mitochondrial protein coding genes, and fibrinogen beta chain intron (*FBG I7*), adenylate kinase 1 intron 5 (*AK1 I5*), ornithine decarboxylase 1 introns 6 and 7 intervening exon (*ODC1*), and Z-linked muscle, skeletal, receptor tyrosine kinase intron 3 (*MUSK I3*) nuclear loci. Partitioning considerably improved mean –lnL values in the Bayesian analyses, with unpartitioned arithmetic mean –lnL = −35,190.36, compared with −34,147.09 for two partitions and −3341.47 for six partitions. Bayes factor comparison also indicated that the 6-partitioned analysis provided better explanations than other data analyses: 2lnB (6-partitions/unpartitioned) = 3697.78, and 2lnB (6-partitions/2-partitions) = 1611.24 significantly above the threshold value of 10. Bayesian posterior probabilities (PP) followed by bootstrap values (ML and MP, respectively) are shown above the branches (only bootstrap values above 50 and PP values above 0.5 are shown for the main clades) for the partitioned analyses. Note that the ID of the only sample of *Selasphorus platycercus* (LSUMNSB23428) included in the phylogeny presented by McGuire et al. [25] is likely incorrect. Painting by Marco Pineda (courtesy of Juan Francisco Ornelas) showing *Calothorax lucifer* (male)

Bayesian analyses using the entire gene data set (nuDNA + mtDNA) resulted in a well-supported phylogeny of the Mellisugini tribe and close relatives (Fig. 1). The summary of MP and ML bootstrap values and the Bayesian posterior probabilities are presented on the branches of the BI 50% majority-rule consensus tree (Fig. 1). Results of other Bayesian analyses using partition or unpartitioned nuDNA or mtDNA data sets are given in Additional file 6. Given the stronger support between clades in the analyses of the nuDNA + mtDNA data set and Bayes Factors, we considered the MrBayes results of concatenated mtDNA + nuDNA genes (with 6-partitions strategy) to be our best estimate of phylogenetic relationships in the Mellisugini. We rely on this tree in our ancestral state reconstructions and discussion of the evolution of migration and of biogeography. Changes to previous phylogenetic topologies of the Mellisugini (e.g. [25]) are indicated in Additional file 7.

### Divergence dating and ancestral areas of bee hummingbirds

**Fig. 2:**
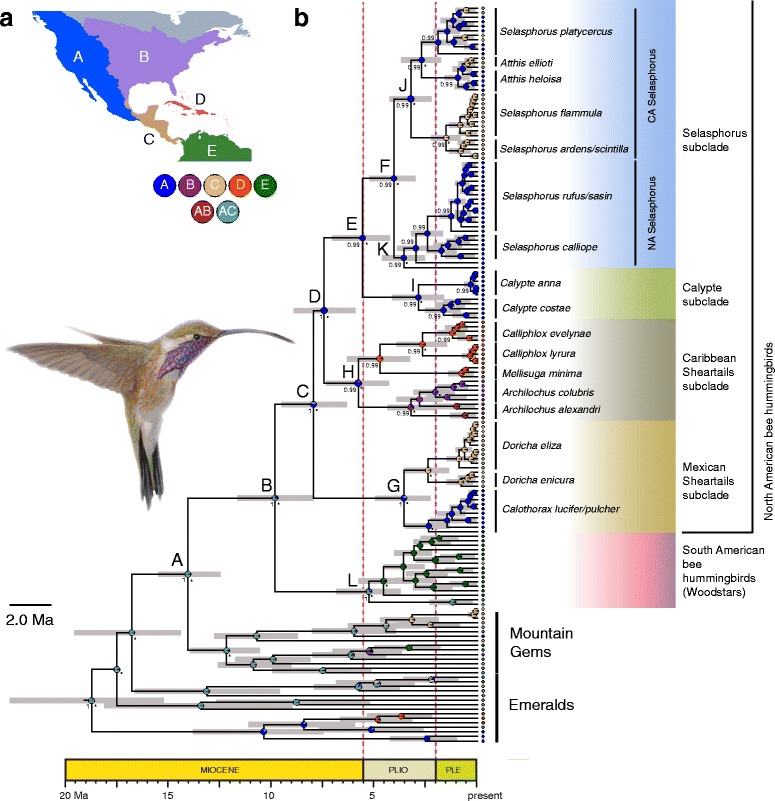
**a** Biogeographic regions used in the RASP analysis: A = western North America, B = eastern North America, C = southeastern Mexico and Central America, D = West Indies, and E = South America. **b** Chronogram of the Mellisugini lineages based on the third calibration method (secondary calibration + substitution rates) with a Yule speciation model for the combined *ND2* and *ND4* mitochondrial genes and *FBG I7*, *AK1 I5*, *ODC1*, and *MUSK I3* nuclear loci data set. Purple bars indicate 95% Highest Posterior Density (HPD) intervals for selected nodes. The pink dotted vertical lines denote the time span of the Pliocene. Results using Bayesian methods with BBM (Bayesian Binary MCMC) analyses implemented in RASP [55] are drawn on this topology. The ancestral origin for each taxon, as delimited in (a), is shown on the terminal lineages. Pie charts at nodes represent the probabilities of the ancestral distributions. These probabilities account for the phylogenetic uncertainty in the rest of the tree and the biogeographic uncertainty at each node. Asterisks next to main nodes refer to posterior probability support for each node (* > 0.8 posterior probability). Painting by Marco Pineda (courtesy of Juan Francisco Ornelas) showing *Calothorax pulcher* (male)

The topology of the BEAST time-tree using the third calibration strategy (secondary calibration + substitution rates (Fig. 2) was concordant with those derived from other reconstruction methods (Fig. 1, Additional file 6). The BEAST analyses indicated the most recent common ancestor (MRCA) for the Mellisugini originated approximately 9.93 MYA (95% HPD 11.94–7.92 MYA) in mainland North America ~6 million years before the final closure of the Isthmus of Panama (Fig. 2). The ancestor originated in either western North America or southern Mexico and Central America, with relatively high support for nodes A (separating bee hummingbirds and mountain gems) and B (separating South and North American bee hummingbirds) yielded by the ancestral area reconstruction (AC, 97% and 79%, respectively; Fig. 2). By the end of the Miocene, the bee hummingbirds first reached western North America (node C; A, 60%). Accordingly, subsequent major nodes (nodes D, E, F, K, J) were reconstructed as nearly 100% western North America (A). Although it is uncertain where the ancestor of bee hummingbirds was distributed in the region, the analysis suggests that western North America was colonized during the early diversification of the group with dispersals into other regions of the Northern Hemisphere. The BEAST analyses also showed a mid-to-late Miocene split separating South American woodstars from the other bee hummingbirds **(**node B; Fig. 2), divergence of the Mexican sheartails from other North American bee hummingbirds in the late Miocene (node C), and that the diversification of the South American woodstars (node L), Caribbean sheartails (node H) and the split between the *Calypte*-*Selasphorus* subclades (node E) occurred in the early Pliocene (Fig. 2). Details of ages for other nodes of interest are shown in Table 1.

**Table 1:** Divergence dates (MYA) of bee hummingbirds for various nodes estimated with a Bayesian uncorrelated lognormal relaxed-clock approach using a Yule speciation tree prior as implemented in BEAST

Node			Several-individuals				Single-individual	
	Secondary calibration + substitution rates		Secondary calibration		Substitution rates		Secondary calibration + substitution rates	
	PP	Mean (95% HPD)	PP	Mean (95% HPD)	PP	Mean (95% HPD)	PP	Mean (95% HPD)
Node A: Bee hummingbirds/\Mountain Gems	1.0	14.08 (15.54–12.51)	1.0	11.83 (13.79–9.85)	1.0	19.97 (24.81–15.64)	0.99	12.49 (13.58–11.37)
Node B: SA/NA bee hummingbirds	1.0	9.93 (11.94–7.92)	0.99	7.68 (9.81–5.60)	1.0	12.50 (16.04–9.11)	1.0	5.92 (6.59–5.24)
Node C: NA crown (Mex. sheartails/other)	1.0	8.03 (9.82–6.44)	0.99	6.32 (8.24–4.51)	1.0	9.56 (12.17–7.05)	1.0	4.98 (5.60–4.41)
Node D: Caribbean Sheartails/other	1.0	7.51 (9.14–5.95)	1.0	5.95 (7.83–4.27)	1.0	8.90 (11.31–6.59)	1.0	4.77 (5.36–4.23)
Node E: *Calypte* subclade/other	0.99	5.72 (7.20–4.31)	0.99	4.12 (5.62–2.73)	1.0	6.71 (9.08–4.87)	0.99	3.11 (3.61–2.62)
Node F: CA *Selasphorus*/NA *Selasphorus*	0.99	4.14 (5.27–3.04)	0.99	3.1 (4.29–1.97)	1.0	4.79 (9.08–4.87)	0.99	2.18 (2.61–1.80)
Node G: Mexican Sheartails crown	1.0	3.61 (5.11–2.39)	0.99	3.09 (4.66–1.70)	1.0	4.39 (6.17–2.71)	1.0	2.20 (2.76–1.69)
Node H: Caribbean sheartails crown	0.99	5.75 (7.35–4.21)	0.99	4.61 (6.21–3.13)	1.0	6.93 (9.28–4.86)	0.99	4.04 (4.61–3.44)
Node I: *Calypte* crown	0.99	3.09 (2.58–0.90)	0.99	2.56 (3.79–1.40)	1.0	3.46 (5.22–1.96)	0.99	2.14 (2.61–1.69)
Node J: CA *Selasphorus* crown	0.99	3.31 (4.42–2.29)	0.99	2.23 (3.21–1.33)	1.0	3.82 (5.12–2.57)	0.99	1.43 (1.77–1.10)
Node K: NA *Selasphorus* crown	0.99	3.66 (4.74–2.60)	0.99	2.64 (1.61–0.48)	1.0	4.24 (5.81–2.86)	0.99	1.16 (1.53–0.79)
Node L: South American crown	1.0	5.42 (7.04–3.81)	0.99	5.44 (7.38–3.67)	1.0	6.44 (8.72–4.32)	1.0	4.30 (4.94–3.65)

**Fig. 3:**
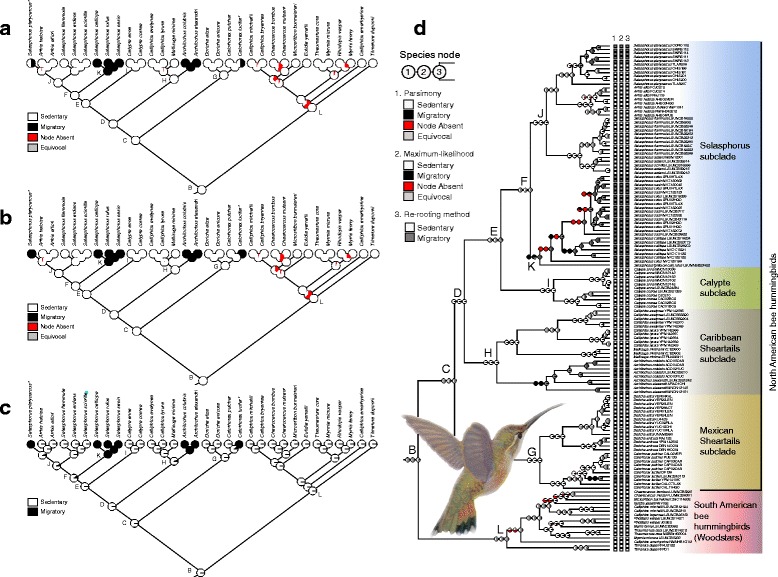
Ancestral state reconstructions across the set of 18,000 post burn-in BEAST trees for the Mellisugini based on parsimony in which species with migratory and sedentary populations (*Selasphorus platycercus* and *Calothorax lucifer*) were coded as polymorphic (**a**) or migratory (**b**), and estimation of ancestral states of migratory behavior carried out with the tree obtained from the Bayesian analysis under the ML criterion and the MK1 model using all samples (**c**). Ancestral state reconstructions results obtained for the MP, ML and re-rooting methods using the data set with several samples (**d**). Each square at the tips of the tree represents the state of each extant taxon and the pie charts at each node represent the probability of the state of the common ancestor present at that node. Dark gray squares indicate fully migrant individuals and white squares indicate sedentary individuals. Asterisks next to species names indicate species with migratory and sedentary populations. Painting by Marco Pineda (courtesy of Juan Francisco Ornelas) showing *Calothorax lucifer* (female)

Posterior probabilities (PP) given for each node; estimates are given as mean ages (in millions of years) with 95% Highest Posterior Density (HPD) intervals in parentheses. The divergence time (mean 12.0 MYA, SD ± 1, range of 13.9–10.3 MYA) between mountain gems and bee hummingbirds [25] was used for temporal calibration of the root node of the tree and the mean substitution rates proposed by Lerner et al. [54] to model the tree prior. Node letters correspond to those in Figs 2– 3. *NA* North America, *SA* South America, *CA* Central America

### Evolution of migratory behavior

The results of ancestral state reconstruction of long-distance migratory behavior on the Bayesian 50% majority-rule consensus tree of a reduced 32 bee hummingbird species data set using only one individual per species and the 6-partitions strategy (Additional file 8) are shown in Fig. 3. The MP and ML reconstructions of migratory behavior provided similar results with high certainty in the bee hummingbirds, in which the phylogenetic position of migratory species indicates multiple independent origin of long-distance migratory behavior (Fig. 3a–c). ML, MP, and the re-rooting method of Yang et al. [67] ancestral-state reconstructions supported a sedentary ancestral mellisuginid (bee hummingbird) regardless of the coding scheme used for migratory behavior (Fig. 3a–c). Because the results using the various coding schemes were largely the same, we only present the results based on one of the coding schemes for each of the ancestral state reconstructions: parsimony and species with partial migration (*C. lucifer* and *S. platycercus*) coded as polymorphic (Fig. 3a), and maximum likelihood and re-rooting with *C. lucifer* and *S. platycercus* coded as migratory (Fig. 3b–c). Basal nodes of all North American subclades (incl. Monophyly of Caribbean sheartails) were reconstructed with strong support for the sedentary state with MP, ML and re-rooting methods (Fig. 3a–c), and migratory behavior was gained four times during the evolution of the Mellisugini: once in the Mexican sheartails subclade (*Calothorax lucifer*) and the Caribbean sheartails subclade (*Archilochus* species), and twice in the *Selasphorus* subclade (*Selasphorus rufus*, *S. sasin* and *S. calliope* group and *S. platycercus*). Similar results were obtained for the MP, ML and re-rooting ancestral state reconstructions using the data set with several samples (Fig. 3d), except that migratory behavior was been lost at least once in *S. platycercus* (see also [37]).

## Discussion

### Phylogeny of bee hummingbirds

Our phylogenetic analyses recovered a monophyletic South American clade sister to other bee hummingbirds in Central America, the Caribbean islands and North America. Despite this, the backbone of our tree topology is not entirely consistent with that for previous studies with fewer taxa and individual samples ([25, 72]; see Additional file 7). According to our analyses, Mexican sheartails split very soon after the split between South American and North American bee hummingbirds, and sister to remaining subclades. The *Selasphorus* subclade is composed of two groups according to our results, the North American *Selasphorus* (*S. calliope*, *S. sasin*, *S. rufus*) and the Central American *Selasphorus* arranged by geography, *S. flammula*, *S. scintilla* and *S. ardens* from Costa Rica and Panama and *S. platycercus*, *Atthis heloisa* and *A. ellioti* mainly from Mexico and Guatemala.

Our DNA sequence data set (including 162 samples and 2–6 loci for each sample) contains 8.8% missing data, and this incompleteness is unlikely to have negatively impacted the accuracy of phylogenetic reconstruction because the number of characters in the analysis is large [73, 74]. Perhaps adding more taxa and more samples per taxon improved the accuracy of our phylogenetic reconstruction, in which monophyly of the Caribbean sheartails (*Archilochus*, *Mellisuga*, *Calliphlox*) is strongly supported. Lastly, our dense individual sampling within *S. platycercus* indicated that this species is nested within the group of Central American *Selasphorus* and *Atthis* species, the *Selasphorus* subclade, and did leverage our phylogenetic and ancestral state reconstructions from ID errors in single-individual species representation of previous phylogenetic reconstructions.

### Divergence dating and ancestral areas of bee hummingbirds

According to our results, the crown age of the Mellisugini, ca. 9.93 MYA using the ‘several-individuals’ data set and the ‘secondary calibration + substitution rates’ calibration strategy, is older than those estimated by McGuire et al. [25] and Abrahamczyk and Renner [72], 5.3 and 7.22–6.71 MYA, respectively. When using the ‘single-individuals’ data set and the ‘secondary calibration + substitution rates’ calibration strategy, the crown age of the Mellisugini is similar, ca. 6.59–5.24 MYA (Table 1). These obvious differences across studies are likely due to the different calibration strategies and taxon sampling employed. McGuire et al. [25] included 27 species and 61 samples of bee hummingbirds and Abrahamczyk and Renner [72] 25 species and 26 samples, whereas our study included 32 species and 132 samples. A discrepancy in age estimates is observed in the comparison of the posterior mean age estimates between the ‘single-individual’ species samples and the ‘several-individuals’ species samples (taxon sampling variation) for the 6-partitions data sets (Table 1). In this case, we know that all sampling and data conditions are identical between the two analyses except for the density of taxon sampling within species. The age estimates for the Mellisugini (nodes B–L) differ on average by about 2 MYA, with the all sampling consistently yielding older ages leading to very different biogeographic conclusions. Although the difference is greater for the deeper nodes in the tree, the impact becomes minor as one moves to the tips of the tree (Fig. 2, Table 1). Our results indicated that different sampling strategies have yielded different estimates and potential errors in molecular dating likely due to sampling bias for recent evolutionary radiations. In discussing age estimates in the subsequent discussion, we refer primarily to the posterior mean point estimated obtained with the ‘several-individuals’ species samples for the 6-partitions data set with more precision in divergence time estimation of shallow nodes.

The early divergences within the Mellisugini are estimated to have occurred after the mid-Miocene climatic optimum (nodes B, C and D of Fig. 2; [75]), but the majority of divergence events occurred much later, from the Pliocene to the mid-late Pleistocene (Fig. 2). Based on these results, we propose that the formation of the mountain systems in Mexico and Central America from mid-Miocene to the Pliocene was critical in providing favorable habitats and climatic conditions for the divergence of bee hummingbirds in the region. The bee hummingbirds dispersed to North America in the mid-Miocene, and then its history was probably marked by a period of expansion to xeric environments and segregation into xeric and moist temperate forests directly associated with a global decrease in temperature and humidity during the Late Miocene [76] and desert formation in North America [77]. Divergences of the Mexican sheartails and the *Calypte* and *Archilochus* species from their ancestors (Fig. 2) dated at 8.03, 5.72 and 5.76 MYA, respectively, coincides with the Miocene peak in speciation rates in some plants characteristic of xeric environments in Mexico, including *Agave* [78] and cacti [79], through a region of the country similar to that through which *Calothorax* is currently distributed and feed upon. The radiation of the Mellisugini in Central America, Caribbean islands and North America must have been relatively rapid. During the Late Miocene, the lineage would already be possibly occupying the main mountain ranges in Central America, and occurring in Mexico, in both montane and dry environments. The divergences of *Calothorax*, *Archilochus*, and the NA *Selasphorus* from their sedentary ancestors are dated at 3.09, 5.75, 4.14 MYA, respectively, suggesting that these divergences occurred in the Pliocene. These divergences also coincide with the second peak in speciation rates in *Agave* sensu *lato* dated at 3–2.5 MYA influenced by nectar feeding bats [78], and the transition from bee-pollination to hummingbird-pollination in Mexico in the *Opuntia-Nopalea* cacti clade dated at 5.73 MYA (95% HPD 8.7–3.42 MYA; [80]). In contrast, divergence of migratory *S. platycercus* from its sedentary ancestor is dated at 1.54 MYA (95% HPD 2.27–0.93 MYA), suggesting that the evolution of migration in *S. platycercus* occurred in the Pleistocene as shown by ancestral state reconstruction (Fig. 3).

The Late Miocene ages of hummingbird-dependent plant clades in North America (9–5 MYA; [72] coincide with the timings of divergence events of bee hummingbirds during the Pliocene and mid-late Pleistocene (Table 1). Our interpretation is also supported by the age of the oldest hummingbird-adapted group in North America, *Lonicera* (Caprifoliaceae), with a stem age of 9.2 MYA and a crown age of 7.0 MYA [81], the age of hummingbird-pollinated *Psittacanthus* mistletoes in Mexico with a stem age of 9.68 MYA and a crown age of 7.43 MYA [82, 83], and by the Pleistocene origin of *Penstemon* in the Rocky Mountains with subsequent migration and radiation to the Cascade–Sierra Nevada cordillera and then into southwestern North America and throughout eastern North America [84]. Interestingly, range expansions of bee hummingbirds in North America during the Pliocene seem to correspond to Pliocene divergences within the hummingbird-pollinated *Psittacanthus* mistletoes apparently linked to habitat shifts [82, 83]. For instance, the ages of the *Calothorax* sheartails, with a stem age of 3.6 MYA (95% HPD 5.11–2.39 MYA) and a crown age of 2.4 MYA (95% HPD 3.61–1.41 MYA), coincide with the timing of divergence events of the *Psittacanthus* mistletoes they currently pollinate (*P. auriculatus* distributed in the xeric areas of Oaxaca and *P. calyculatus* distributed in pine-oak forests along the Trans-Mexican Volcanic Belt; [85–87]), with a stem age of 3.1 MYA and a crown age of 1.8 MYA [82, 83]. The ages of *Calypte costae*, with a stem age of 3.1 MYA (95% HPD 4.49–1.78 MYA) and a crown age of 1.7 MYA (95% HPD 2.58–0.91 MYA), coincide with the timing of divergence events of the *Psittacanthus* mistletoes they currently pollinate in the Sonoran Desert, *P. sonorae*, with a stem age of 4.8 MYA and a crown age of 0.3 MYA [82, 83].

Like the hummingbirds and their coevolved plants in North America, the earliest divergence within the Caribbean sheartails (5.7 MYA; Table 1) indicates that they were also contemporaneous with lineages of hummingbird-adapted flowers [72, 88]. Overall, the results of our divergence-dating analysis seem to indicate that the Pliocene range expansions of bee hummingbirds are connected with the biogeography of their host plants and provide interesting insights on how range expansions into North America via habitat changes facilitated the evolution of migration in this group.

### Evolution of migratory behavior

Our study is the first to provide phylogenetic evidence for the repeated evolution of long-distance migratory behavior in the radiation of the Mellisugini, with the crown ancestors of the main clades and North American subclades reconstructed as sedentary. Our results suggest that long-distance seasonal migration arose independently four times in the Mellisugini: once in the Mexican sheartails subclade (*Calothorax lucifer*) and the Caribbean sheartails subclade (*Archilochus* species), and twice in the *Selasphorus* subclade (*Selasphorus rufus*, *S. sasin* and *S. calliope* group and *S. platycercus*). Our study also showed that migratory lineages are generally more closely related to non-migratory lineages than to other migratory lineages, and that long-distance seasonal migration arose at different times. For instance, the split between migratory *Archilochus* species and sedentary species endemic to the Caribbean islands (*Mellisuga* and *Calliphlox* species) occurred at 5.7 MYA, whereas genetic differentiation between migratory *C. lucifer* and *S. platycercus* and their sedentary ancestors seem to have started during the late Pleistocene.

Our ancestral area reconstruction appears to explain the migration patterns of earliest *Archilochus* species, earliest *A. colubris* breeding in eastern NA (USA and Canada) and migrant to mainly eastern Mexico, Central America and some Caribbean islands, and *A. alexandri* breeding in western NA (USA and Canada) and migrant to western Mexico. A phylogeographic approach accompanied with paleodistributional modeling would be needed to test whether the differences in migratory patterns between *Archilochus* sister species were influenced by Pleistocene climate change and their range shifts occurred earlier. Using ecological niche modeling and phylogeographic data, Malpica and Ornelas [37] showed first that *S. platycercus* is a niche tracker and then that the climate conditions associated with modern obligate migrants in the USA were not present during the LIG, which provides indirect evidence for recent migratory behavior in *S. platycercus* on the temporal scale of glacial cycles. Their study also revealed that the evolution of migration within *S. platycercus* produced no significant genetic structure using nuclear microsatellites (nSSRs), migratory and sedentary groups of populations form an admixed population. The fact that they detected no significant genetic differentiation between migratory populations of *S. platycercus* and sedentary populations of the species in central Mexico (*platycercus* subspecies) is surprising because these hummingbirds inhabit different breeding areas of the USA and Mexico, and no evidence of sympatry at overwintering sites in Mexico has been noted. However, phylogeographic analyses and population genetic methods revealed that both migratory populations in the USA and sedentary populations in Mexico of the *platycercus* subspecies form one admixed population, and that sedentary populations from southern Mexico and Guatemala (*guatemalae* subspecies) diverged earlier (0.75 MYA) and undertook independent evolutionary trajectories [37].

Several studies have explicitly outlined a similar framework for addressing the evolution of bird migration between North and Central America, in particular for species with migratory populations in Canada and USA and sedentary populations in Mexico and Central America [10, 89]. For example, Milá et al. [90] examined the evolution of migration in the chipping sparrow (*Spizella passerina*) with sedentary populations in Mexico and the southern USA and migratory populations in the northern USA and Canada, and found evidence that migration was driven by northern range expansion from sedentary populations following glacial episodes.

Our study provides phylogenetic evidence for a sedentary origin for the Mellisugini, but certainly is not the first to deal with this question in birds of the Northern Hemisphere (e.g., [60, 89–98]). Studies that have reconstructed the ancestral state of migration in a phylogenetic context have found either equivocal results [93, 99, 100] or results in favor of a migratory [10, 60, 101] or a sedentary ancestor [89, 92, 98, 102–104]. According to a well-supported molecular phylogeny of *Catharus* thrushes *sensu lato* (incl. *Hylocichla mustelina*), long-distance seasonal migration is reconstructed as the ancestral condition at most basal nodes when putting character changes as close to the root of the tree as possible (ACCTRAN resolving option), and north of Mexico is reconstructed as the ancestral area with the origin of the clade at 8 MYA, diversification of *Catharus* from *Hylocichla* occurring at 6.9 MYA, and further lineage divergence within *Catharus* starting in the early Pliocene at 4.7 MYA [10]. Within *Catharus*, migratory behavior was lost after the first speciation event in the genus and was geographically and temporally correlated with Central American distributions and the final closure of the Central American Seaway. Subsequently, migratory behavior was re-gained twice in *Catharus* and was geographically and temporally correlated with a re-colonization of North America in the late Pleistocene [10]. Counter to our results for the Mellisugini, the ancestral wood-warbler (Parulidae) was reconstructed as migratory using the well-supported molecular phylogeny of Lovette et al. [97], with losses of migration as prevalent as gains throughout the evolutionary history of Parulidae [60]. These results suggest that extant sedentary tropical radiations in the Parulidae represent losses of long-distance seasonal migration and colonization of the tropics from temperate regions [60]. However, many derived non-migratory clades descended from non-migratory ancestors, supporting the notion that the ancestor of the Parulidae was a non-migrant [98]. Using a phylogenetic model of the joint evolution of breeding and non-breeding, wintering ranges to infer the biogeographic history in the emberizoid passerine birds, Winger et al. [101] found that seasonal migration between breeding ranges in North America and winter ranges in the Neotropics evolved primarily via shifts of winter ranges toward the tropics from ancestral ranges in North America.

In the Mellisugini, it seems that migration evolved out of the tropics through the northern extension of ancestral tropical or subtropical breeding ranges into temperate regions (‘southern home-theory’; [6, 9]). According to results of the Rolland et al.’s [8] study that included most extant bird species, we infer that sedentary behavior is ancestral and migratory behavior evolved several times during the evolutionary history of the Mellisugini. Testing increased diversification rates in the Mellisugini with the evolution of migration is hampered by the lack of statistical power (see [8] for further discussion). Nonetheless, the divergence of a migratory species into two migratory daughter species tend to be less frequent that the divergence of a sedentary species into two sedentary daughter species, consistent with the findings of Rolland et al. [8] and predictions of Helbig [5] and Claramunt et al. [105] that genetic differentiation is reduced in migratory species with high dispersal capacity. The results of Rolland et al.’s [8] study suggest that the mobility of migratory species promotes the colonization of new areas and, if adapted to the new habitat, populations can become sedentary and diverge from the founding migratory species.

Several factors would have influenced the way bee hummingbirds colonized the northern portion of North America in the mid-Pliocene. Following the first dispersal of ancestral *Archilochus* hummingbirds from the Caribbean islands to the northeast and northwest of North America, either along the coastal slope or across the Gulf of Mexico, it is possible that range changes in the Pleistocene caused multiple populations to lose migration and stay restricted to the Caribbean Islands with subsequent speciation. Successful dispersal of North American *Selasphorus* hummingbirds occurred from Central America and southern Mexico to the northwest of the continent. The evolution of migratory populations from ancestral sedentary populations in southern Mexico of *S. platycercus* occurred later, likely due to Pleistocene climate changes (see also [90]). These scenarios are consistent with the idea that geographic isolation during the Late Pleistocene account for intraspecific and sister-species-level divergence largely based on habitat shifts influenced by climate change (e.g., [102, 106–108]), particularly shifts subsequent to the LGM produced distinct migratory pathways and further genetic differentiation [93, 109]. Therefore, it seems that divergence of a migratory species into two migratory daughter species is linked to a seemly rare event of changing migratory trajectories widely documented in some songbirds (e.g., [93, 109–116]). Further study using a comparative phylogeographic approach accompanied with ecological niche modeling and more or faster molecular markers (e.g., SNPs, SSRs) should provide finer resolution to the history of migration in the Mellisugini, particularly for contrasts between shallow lineages with sedentary and migratory species (e.g., *Calothorax lucifer*/*C. pulcher*). For testing the effects of cyclical glacial changes on producing seasonally unstable habitats, and driving northward expansion and the evolution of seasonal migration and contraction into southern sedentary populations, and increased sampling of South American woodstars, further study will be needed to test whether the evolution of long-distance seasonal migration in North American bee hummingbirds has facilitated diversification in the Mellisugini through the divergence of migratory subpopulations that become sedentary [8, 60].

We cannot eliminate the possibility that long-distance migratory behavior evolved relatively early in the evolution of the Mellisugini. If this was the case, a migratory ancestor lost migration multiple times. Under this hypothesis colonization of North America and the Caribbean Islands would be more likely, despite that the phylogenetic evidence of that migratory ancestor is now lost, as temperate niches remained relatively open with ephemeral resources with subsequent losses of migratory at later times environments became less seasonal. If this model were statistically supported, its results would suggest that long-distance migratory behavior evolved once in the base of the Mellisugini tree, with several subsequent losses towards the end of the Pliocene. In coding SA bee hummingbirds and mountain-gems as migratory assuming that a migratory ancestor lost migration multiple times, ancestral character state reconstruction yielded equivocal results for the node of the Mellisugini and further ancestral nodes within the tribe were reconstructed as sedentary. When forcing only SA bee hummingbirds to be migratory, both the node of the Mellisugini and further ancestral nodes for main clades within the tribe were reconstructed as sedentary (Results not shown).

Cox’s [11] model predicts that migratory species will be derived from sedentary species within the seasonal subtropics, and that migratory behavior is a derived character state. We believe that the Mellisugini lineage fits this model in many ways, particularly because most migrant species are closely related to the sedentary species found in the seasonal highlands of Mexico and Central America. An important result of our study is that long-distance migratory species do not form a monophyletic group. The relationships between migrant and resident species within the Mexican sheartails, Caribbean sheartails, and *Selasphorus* subclades are more complex than we expected. Also, the repeated gains of migration occurred during the Late Pliocene and this suggests that potential responses, i.e. the temporal evolution of migratory behavior, can be linked to historical, climatic and ecological events on a phylogeny [7, 10]. However, we cannot ignore the possibility that long-distance migratory behavior in the Mellisugini was the ancestral state with several drop-offs of migration. Given the high degree of lability of the trait [34–36] and assuming that the phylogenetic signal of long-distance migratory behavior in the Mellisugini is an artifact of phylogenetic inertia in biogeographic range (including latitude and temperature seasonality), these questions seem unanswerable, making long-distance seasonal migration non tractable over substantial evolutionary time until comparative genomic data sets for migratory/sedentary closely related species pairs and for migratory and non-migratory populations of species with partial migration become available.

## Conclusions

Pliocene’s mountain building in Mexico and Pleistocene climate changes were the primary feature that structured diversity in the Mellisugini. These results are consistent with Cox’s [12] idea that the Mexican Plateau and arid southwestern United States have acted as staging areas for the evolution of hummingbird migration. Range expansions of early lineages of the Mellisugini seem to be connected with the biogeography of their host plants and provide interesting insights on how range expansions into North America via habitat changes facilitated the evolution of migration in this group. Recently evolved lineages in all subclades of the Mellisugini appear to have undergone long-distance seasonal migration, albeit in different directions. This history of repeated evolution of migration within the Mellisugini allowed for divergence across common biogeographic regions spanned by North American bee hummingbirds. It is likely that, without repeated evolution of migration in different directions, diversification of the Mellisugini would have decelerated towards the present [25]. Thus, molecular patterns of diversification within the Mellisugini reflect a dynamic history of divergence, the main lineages during the Pliocene linked to the formation of the mountain systems in Mexico and Central America and further divergence by the evolution of seasonal migration during the Pleistocene.

## Additional files

Additional file 1: Primers employed in this study. (DOC 39 kb)

Additional file 2: Species names, voucher information, locality, and GenBank accession numbers for specimens sequenced in this study. (DOC 102 kb)

Additional file 3: Species names, distributional codes and migratory status for ancestral state reconstruction analyses of the Mellisugini species used in this study. A = western North America, B = eastern North America, C = eastern Mexico and Central America, D = West Indies, E = South America; M = migratory, S = sedentary (binary character codification). (DOC 169 kb)

Additional file 4: Migratory status of the Mellisugini species for ancestral state reconstruction analysis used in this study. M = migratory, S = sedentary. (DOC 57 kb)

Additional file 5: Species names, English names and distributional range for the Mellisugini species used in this study. (DOC 75 kb)

Additional file 6: Bayesian 50% majority rule consensus trees of 132 representatives of bee hummingbirds (32 of the 36 extant species, 89%), 15 of mountain gems and 15 of emeralds. The trees are based on data sets of (a) only mitochondrial genes (‘unpartitioned mtDNA data set’), (b) only mitochondrial genes as two partitions (“partitioned mtDNA data set”), (c) only nuclear genes (‘unpartitioned nuDNA data set”), and (d) only nuclear genes as four partitions (“partitioned nuDNA data set”). Posterior probabilities (PP) > 0.5 are shown. (PDF 930 kb)

Additional file 7: Comparison of backbone tree topologies of the Mellisugini. (a) McGuire et al. [25], (b) Abrahamczyk & Renner [72], and (c) Bayesian 50% majority rule consensus tree of 32 bee hummingbird species of this study in Additional file 7. Asterisks denote nodes with 1.0 posterior probability (PP) support. Numbers at nodes reflect posterior probabilities less than 1.0. Support values for nodes of phylogeny in (b) are not provided in Abrahamczyk & Renner [72]. (PDF 425 kb)

Additional file 8: Bayesian 50% majority rule consensus tree of 32 bee hummingbird species and representatives of mountain gems and emeralds used as outgroups. The tree is based on a combined data set of all available fragments of *ND2*, *ND4*, *AK1 I5*, *MUSK I3*, *ODC1* and *FBG I7* and partition-specific DNA evolution models of each gene (‘6-partitions data set’). Posterior probabilities (PP) > 0.5 are shown. (PDF 404 kb)
