# Supplementary material for: The conquering of North America: dated phylogenetic and biogeographic inference of migratory behavior in bee hummingbirds
Source: BMC Evol Biol. 2017 Jun 5;17:126. doi: 10.1186/s12862-017-0980-5 (PMC5460336; doi:10.1186/s12862-017-0980-5)
Supplement: Supplementary file 1 — Primers employed in this study. (DOC 39 kb) [file 12862_2017_980_MOESM1_ESM.doc]

**Additional file 2** Primers employed in this study.

| Locus | Primer name | Primer sequence 5'- 3' |
| --- | --- | --- |
| *ND2* | L5216 [1] | GGCCCATACCCCGRAAATG |
| *ND2* | H5766 [1] | GGATGAGAAGGCTAGGATTTT KCG |
| *ND2* | L5758 [1] | GGCTGAATRGGMCTNAAYCARAC |
| *ND2* | H6313 [1] | CTCTTATTTAAGGCTTTGAAAGGC |
| *ND4* | L12156 [2] | CACCTATGACTACCAAAAGCTCATGTAGAAGC |
| *ND4* | H13047 (ND4Leu) [2] | CATTACTTTTACTTGGATTTGCACCA |
| *FGB I7* | FIB-B17U [3] | GGAAAAACAGGACAATGACAATTCAC |
| *FGB I7* | FIB-B17L [3] | TCCCCAGTAGTATCTGCCATTAGGGTT |
| *AK1 I5* | AK5b-inset [4] | GGCTACCCTCGCGAGGTGAAACAG |
| *AK1 I5* | AK5b-inset [4] | TGGTCTCTCCTCGCTTCAG |
| *ODC1* | ODC2-F [5] | GCGTGCAAAAGAACTTGACC |
| *ODC1* | ODC2-R [5] | AGCCACCACCAATATCAAGC |
| *MUSK I3* | MUSK-F3 [6] | GCTGTACTTCCATGCACTACAATG |
| *MUSK I3* | MUSK-R3 [6] | ATCCTCAAATTTCCCGAATCAAG |

**References**

1. Sorenson MD, Ast JC, Dimcheff DE, Yuri T, Mindell DP. Primers for a PCR-based approach to mitochondrial genome sequencing in birds and other vertebrates. Molecular Phylogenetics and Evolution 1999;12(2):105–114.
2. Arévalo E, Davis SK, Sites JW. Mitochondrial DNA sequence divergence and phylogenetic relationships among eight chromosome races of the *Sceloporus grammicus* complex (Phrynosomatidae) in central Mexico. Systematic Biology 1994;43(3):387–418.
3. Prychitko TM, Moore WS. The utility of DNA sequences of an intron from the b-fibrinogen gene in phylogenetic analysis of woodpeckers (Aves: Picidae). Molecular Phylogenetics and Evolution 1997;8(2):193–204.
4. McGuire JA, Witt CC, Altshuler DL, Remsen JV. Phylogenetic systematics and biogeography of hummingbirds: Bayesian and maximum likelihood analyses of partitioned data and selection of an appropriate partitioning strategy. Systematic Biology 2007;56(5):837–856.
5. Parra JL, Remsen JV, Alvarez-Rebolledo M, McGuire JA. Molecular phylogenetics of the hummingbird genus *Coeligena*. Molecular Phylogenetics and Evolution 2009;53(2):425–434.
6. Benham PM, Cuervo AM, McGuire JA, Witt CC. Biogeography of the Andean metaltail hummingbirds: contrasting evolutionary histories of tree line and habitat generalist clades. Journal of Biogeography 2015;42(4):763–777.
