# Supplementary material for: The conquering of North America: dated phylogenetic and biogeographic inference of migratory behavior in bee hummingbirds
Source: BMC Evol Biol. 2017 Jun 5;17:126. doi: 10.1186/s12862-017-0980-5 (PMC5460336; doi:10.1186/s12862-017-0980-5)
Supplement: Supplementary file 2 — Species names, voucher information, locality, and GenBank accession numbers for specimens sequenced in this study. (DOC 102 kb) [file 12862_2017_980_MOESM2_ESM.doc]

**Additional file 1** Species names, voucher information, locality, and GenBank accession numbers for specimens sequenced in this study.

|  |  |  |  | GenBank accession numbers | | | | | |  |
| --- | --- | --- | --- | --- | --- | --- | --- | --- | --- | --- |
| Species | Museum | Voucher number | Locality | *ND2* | *ND4* | *AK1 I5* | *MUSK I3* | *ODC1* | *BFG I7* | |
|  |  |  |  |  |  |  |  |  |  | |
| *Archilochus alexandri* | INECOL | ARAO1CHI | MX: Chihuahua | KX855335 | KX855394 | KX855486 | KX855569 | KX855510 | - | |
| *Archilochus alexandri* | MNCN | 13-145 | USA: CA, Tuna Canyon | KX855336 | KX855395 | KX855487 | KX855568 | KX855511 | KX855627 | |
| *Archilochus alexandri* | MNCN | 13-151 | USA: CA, Tuna Canyon | KX855337 | KX855396 | KX855488 | KX855570 | KX855512 | KX855628 | |
| *Archilochus colubris* | INECOL | ACO02YUC | MX: Yucatán | KX855338 | KX855397 | KX855451 | KX855571 | KX855513 | - | |
| *Archilochus colubris* | INECOL | ACO03YUC | MX: Yucatán | KX855339 | KX855398 | KX855489 | KX855572 | KX855514 | - | |
| *Archilochus colubris* | INECOL | ACO04OAX | MX: Oaxaca, Cerro Baúl | KX855340 | KX855399 | KX855490 | KX855573 | KX855515 | - | |
| *Archilochus colubris* | INECOL | ACO05OAX | MX: Oaxaca, Cerro Baúl | KX855341 | KX855400 | KX855452 | KX855574 | KX855516 | - | |
| *Atthis heloisa* | INECOL | AHE02VER | MX: Veracruz | KX855345 | KX855404 | KX855456 | KX855578 | KX855520 | - | |
| *Atthis heloisa* | INECOL | AHE03HGO | MX: Hidalgo, Oxpantla | KX855346 | KX855405 | KX855457 | KX855579 | KX855521 | - | |
| *Atthis heloisa* | INECOL | AHE04PUE | MX: Puebla, Lagunillas | KX855347 | KX855406 | KX855468 | KX855580 | KX855522 | - | |
| *Atthis ellioti* | MCUSC | RAJ179 | Guatemala | KX855344 | KX855403 | KX855455 | KX855577 | KX855519 | KX855626 | |
| *Atthis ellioti* | MCUSC | CJC274 | Guatemala | KX855343 | KX855402 | KX855454 | KX855576 | KX855518 | KX855625 | |
| *Atthis ellioti* | MCUSC | CJC272 | Guatemala | KX855342 | KX855401 | KX855453 | KX855575 | KX855517 | - | |
| *Calothorax lucifer* | INECOL | DF136 | MX: DF, El Pedregal | KX855348 | KX855407 | KX855471 | KX855581 | KX855523 | - | |
| *Calothorax lucifer* | INECOL | CAL07TLAX | MX: Tlaxcala, Tecoac | KX855350 | KX855408 | KX855474 | KX855582 | KX855524 | - | |
| *Calothorax lucifer* | INECOL | CAL11HGO | MX: Hidalgo, Cuautepec | KX855351 | KX855409 | KX855475 | - | KX855525 | - | |
| *Calothorax pulcher* | INECOL | PUE135 | MX: Puebla, Tehuacán | KX855355 | KX855414 | KX855470 | KX855587 | KX855530 | - | |
| *Calothorax pulcher* | INECOL | CAL02VER | MX: Veracruz, Perote | KX855349 | KX855410 | KX855472 | KX855583 | KX855526 | - | |
| *Calothorax pulcher* | INECOL | CAP02OAX | MX: Oaxaca, San Baltazar | KX855352 | KX855411 | KX855476 | KX855584 | KX855527 | - | |
| *Calothorax pulcher* | INECOL | CAP03OAX | MX: Oaxaca, Matatlán | KX855353 | KX855412 | KX855477 | KX855585 | KX855528 | - | |
| *Calothorax pulcher* | INECOL | CAP04OAX | MX: Oaxaca, Matatlán | KX855354 | KX855413 | KX855473 | KX855586 | KX855529 | - | |
| *Calypte anna* | MNCN | 13-036 | MX: BCN, Sn Pedro Mártir | KX855356 | KX855415 | KX855491 | KX855588 | KX855531 | KX855629 | |
| *Calypte anna* | MNCN | 13-108 | MX: BCN, Sn Pedro Mártir | KX855357 | KX855416 | KX855492 | KX855589 | KX855532 | KX855630 | |
| *Calypte anna* | MNCN | 13-142 | USA: CA, Tuna Canyon | KX855358 | KX855417 | KX855493 | KX855590 | KX855533 | KX855631 | |
| *Calypte anna* | MNCN | 13-143 | USA: CA, Tuna Canyon | KX855359 | KX855418 | KX855494 | KX855591 | KX855534 | KX855632 | |
| *Calypte anna* | MNCN | 13-153 | USA: CA, Kern River Preserve | KX855360 | KX855419 | KX855495 | KX855592 | KX855535 | KX855633 | |
| *Calypte costae* | CIBNOR | CAC01BCS | MX: BCS, Santa Gertrudis | KX855361 | KX855420 | KX855496 | KX855593 | KX855536 | - | |
| *Calypte costae* | CIBNOR | CAC02BCS | MX: BCS, Santa Gertrudis | KX855362 | - | KX855497 | KX855594 | KX855537 | KX855635 | |
| *Calypte costae* | CIBNOR | CAC03BCS | MX: BCS, Santa Gertrudis | KX855363 | KX855421 | KX855498 | KX855595 | KX855538 | KX855634 | |
| *Doricha eliza* | INECOL | VER01LEN | MX: Veracruz, El Lencero | KX855364 | KX855422 | KX855469 | KX855596 | KX855539 | - | |
| *Doricha eliza* | INECOL | VER04XAL | MX: Veracruz, Xalapa | KX855365 | KX855423 | KX855478 | KX855597 | KX855540 | KX855636 | |
| *Doricha eliza* | INECOL | VER23LEN | MX: Veracruz, El Lencero | KX855366 | KX855424 | KX855479 | - | - | - | |
| *Doricha eliza* | INECOL | VER24LEN | MX: Veracruz, El Lencero | KX855367 | KX855425 | KX855480 | KX855598 | KX855541 | - | |
| *Doricha eliza* | INECOL | VER25LEN | MX: Veracruz, El Lencero | KX855368 | KX855426 | KX855481 | KX855599 | KX855542 | - | |
| *Doricha eliza* | INECOL | VER26ACT | MX: Veracruz, Actopan | KX855369 | KX855427 | KX855482 | KX855600 | KX855543 | - | |
| *Doricha eliza* | INECOL | YUC09RLA | MX: Yucatán, Río Lagartos | KX855370 | KX855428 | KX855483 | KX855601 | KX855544 | - | |
| *Doricha eliza* | INECOL | YUC10RLA | MX: Yucatán, Río Lagartos | KX855371 | KX855429 | KX855484 | KX855602 | KX855545 | - | |
| *Doricha eliza* | INECOL | YUC18CHI | MX: Yucatán, Chicxulub | KX855372 | KX855430 | KX855485 | KX855603 | KX855546 | - | |
| *Doricha enicura* | INECOL | DEN14COM | MX: Chiapas, Comitán | KX855373 | KX855431 | KX855458 | KX855604 | KX855547 | - | |
| *Doricha enicura* | MCUSC | RAJ182 | Guatemala | KX855375 | KX855433 | KX855460 | KX855606 | KX855549 | KX855637 | |
| *Doricha enicura* | INECOL | DEN16COM | MX: Chiapas, Comitán | KX855374 | KX855432 | KX855459 | KX855605 | KX855548 | - | |
| *Selasphorus platycercus* | INECOL | CORO102 | USA: AZ, Coronado Ranch | KX855380 | KX855438 | KX855499 | KX855611 | KX855554 | - | |
| *Selasphorus platycercus* | INECOL | SWRS111 | USA: AZ, SW Research Station | KX855381 | KX855439 | KX855500 | KX855612 | KX855555 | - | |
| *Selasphorus platycercus* | INECOL | SWRS112 | USA: AZ, SW Research Station | KX855382 | KX855440 | KX855501 | KX855613 | KX855556 | - | |
| *Selasphorus platycercus* | INECOL | SWRS113 | USA: AZ, SW Research Station | KX855383 | KX855441 | KX855502 | KX855614 | KX855557 | - | |
| *Selasphorus platycercus* | INECOL | SWRS114 | USA: AZ, SW Research Station | KX855384 | KX855442 | KX855503 | KX855615 | KX855558 | - | |
| *Selasphorus platycercus* | INECOL | CHIS197 | USA: AZ, SW Research Station | KX855376 | KX855434 | KX855461 | KX855607 | KX855550 | - | |
| *Selasphorus platycercus* | INECOL | CHIS199 | USA: AZ, SW Research Station | KX855377 | KX855435 | KX855462 | KX855608 | KX855551 | - | |
| *Selasphorus platycercus* | INECOL | CHIS200 | USA: AZ, SW Research Station | KX855378 | KX855436 | KX855463 | KX855609 | KX855552 | - | |
| *Selasphorus platycercus* | INECOL | CHIS201 | USA: AZ, SW Research Station | KX855379 | KX855437 | KX855464 | KX855610 | KX855553 | - | |
| *Selasphorus platycercus* | INECOL | TLAX206 | MX: Tlaxcala, La Cueva | KX855385 | KX855443 | KX855465 | KX855616 | KX855559 | - | |
| *Selasphorus platycercus* | INECOL | TLAX207 | MX: Tlaxcala, La Cueva | KX855386 | - | KX855466 | KX855617 | KX855560 | - | |
| *Selasphorus rufus* | INECOL | SRU01HGO | MX: Hidalgo, Mineral de la R | KX855387 | KX855444 | KX855504 | KX855618 | KX855561 | - | |
| *Selasphorus rufus* | INECOL | SRU02HGO | MX: Hidalgo, Mineral de la R | KX855388 | KX855445 | KX855505 | KX855619 | KX855562 | - | |
| *Selasphorus rufus* | INECOL | SRU03HGO | MX: Hidalgo, Mineral de la R | KX855389 | KX855446 | KX855506 | KX855620 | KX855563 | - | |
| *Selasphorus rufus* | INECOL | SRU04TLAX | MX: Tlaxcala, San Francisco | KX855390 | KX855447 | KX855507 | KX855621 | KX855564 | - | |
| *Selasphorus rufus* | INECOL | SRU05TLAX | MX: Tlaxcala, San Francisco | KX855391 | KX855448 | KX855508 | KX855622 | KX855565 | - | |
| *Selasphorus rufus* | INECOL | SRU06TLAX | MX: Tlaxcala, San Francisco | KX855392 | KX855449 | KX855509 | KX855623 | KX855566 | - | |
| *Tilmatura dupontii* | INECOL | PD-1 | MX: Chiapas, Monte Sinaí | KX855393 | KX855450 | KX855467 | KX855624 | KX855567 | - | |
|  |  |  |  |  |  |  |  |  |  | |

Museum abbreviations: INECOL = Instituto de Ecología, AC, Xalapa, Veracruz, Mexico; MCUSC = Museo Facultad de Biología, Universidad San Carlos, Guatemala; CIBNOR = Centro de Investigación Biológica del Noreste, La Paz, Baja California Sur, Mexico; MNCN = Museo Nacional de Ciencias Naturales, Madrid, Spain.
