# Supplementary material for: The conquering of North America: dated phylogenetic and biogeographic inference of migratory behavior in bee hummingbirds
Source: BMC Evol Biol. 2017 Jun 5;17:126. doi: 10.1186/s12862-017-0980-5 (PMC5460336; doi:10.1186/s12862-017-0980-5)
Supplement: Supplementary file 3 — Species names, distributional codes and migratory status for ancestral state reconstruction analyses of the Mellisugini species used in this study. A = western North America, B = eastern North America, C = eastern Mexico and Central America, D = West Indies, E = South America; M = migratory, S = sedentary (binary character codification). (DOC 169 kb) [file 12862_2017_980_MOESM3_ESM.doc]

**Additional file 3** Species names, distributional codes and migratory status for ancestral state reconstruction analyses of the Mellisugini species used in this study: A = western North America, B = eastern North America, C = eastern Mexico and Central America, D = West Indies, E = South America; M = migratory, S = sedentary (binary character codification).

| Species | Distribution range | Migratory status |
| --- | --- | --- |
| *Archilochus alexandri* ARA01CHI | AB | M |
| *Archilochus alexandri* LSUMZB21848 | AB | M |
| *Archilochus alexandri* MNCNBCHU13145 | AB | M |
| *Archilochus alexandri* MNCNBCHU13151 | AB | M |
| *Archilochus colubris* ACO02YUC | B | M |
| *Archilochus colubris* ACO03YUC | B | M |
| *Archilochus colubris* ACO04OAX | B | M |
| *Archilochus colubris* ACO05OAX | B | M |
| *Archilochus colubris* LSUMZB5270 | B | M |
| *Atthis ellioti* CJC272 | C | S |
| *Atthis ellioti* CJC274 | C | S |
| *Atthis ellioti* RAJ179 | C | S |
| *Atthis heloisa* AHE02VER | A | S |
| *Atthis heloisa* AHE03HGO | A | S |
| *Atthis heloisa* AHE04PUE | A | S |
| *Atthis heloisa* FMNH343218 | A | S |
| *Atthis heloisa* UNAMOVMP1041 | A | S |
| *Calliphlox amethystina* NMNHb10703 | E | S |
| *Calliphlox bryantae* LSUMZB28180 | C | S |
| *Calliphlox evelynae* LSUMZ59204 | D | S |
| *Calliphlox evelynae* LSUMZB58890 | D | S |
| *Calliphlox evelynae* YPM142569 | D | S |
| *Calliphlox evelynae* YPM142570 | D | S |
| *Calliphlox evelynae* YPM142568 | D | S |
| *Calliphlox lyrura* YPM142562 | D | S |
| *Calliphlox lyrura* YPM142564 | D | S |
| *Calliphlox lyrura* YPM142565 | D | S |
| *Calliphlox lyrura* YPM142566 | D | S |
| *Calliphlox lyrura* YPM142567 | D | S |
| *Calliphlox mitchellii* LSUMZB12194 | E | S |
| *Calliphlox mitchellii* LSUMZB2310 | E | S |
| *Calothorax lucifer* DF136 | A | S |
| *Calothorax lucifer* CAL07TLAX | A | S |
| *Calothorax lucifer* CAL11HGO | A | S |
| *Calothorax lucifer* LSUMZB43113 | A | M |
| *Calothorax lucifer* YPM141067 | A | M |
| *Calothorax pulcher* CAL02VER | A | S |
| *Calothorax pulcher* CAP02OAX | A | S |
| *Calothorax pulcher* CAP03OAX | A | S |
| *Calothorax pulcher* CAP04OAX | A | S |
| *Calothorax pulcher* PUE135 | A | S |
| Calypte *anna* LSUMZB24864 | A | S |
| *Calypte anna* MNCN13036 | A | S |
| *Calypte anna* MNCN13108 | A | S |
| *Calypte anna* MNCN13142 | A | S |
| *Calypte anna* MNCN13143 | A | S |
| *Calypte anna* MNCN13153 | A | S |
| *Calypte costae* CAC01BCS | A | S |
| *Calypte costae* CAC02BCS | A | S |
| *Calypte* *costae* CAC03BCS | A | S |
| *Calypte costae* CJC210 | A | S |
| *Calypte costae* LSUMZB21595 | A | S |
| *Chaetocercus bombus* LSUMZB5225 | E | S |
| *Chaetocercus mulsant* LSUMZB6301 | E | S |
| *Doricha eliza* KU4435 | C | S |
| *Doricha eliza* UNAMb590 | C | S |
| *Doricha eliza* VER01LEN | C | S |
| *Doricha eliza* VER04XAL | C | S |
| *Doricha eliza* VER23LEN | C | S |
| *Doricha eliza* VER24LEN | C | S |
| *Doricha eliza* VER25LEN | C | S |
| *Doricha eliza* VER26ACT | C | S |
| *Doricha eliza* YUC09RLA | C | S |
| *Doricha eliza* YUC10RLA | C | S |
| *Doricha eliza* YUC18CHI | C | S |
| *Doricha enicura* DEN14COM | C | S |
| *Doricha enicura* DEN16COM | C | S |
| *Doricha enicura* RAJ182 | C | S |
| *Doricha enicura* YPM142508 | C | S |
| *Eulidia yarrellii* WV022 | E | S |
| *Mellisuga minima* MVZ183600 | D | S |
| *Mellisuga minima* MVZ183602 | D | S |
| *Mellisuga minima* STRIJAMM11 | D | S |
| *Microstilbon burmeisteri* ZMC114832 | E | S |
| *Myrmia micrura* LSUMZB5233 | E | S |
| *Myrtis fanny* LSUMNSB3592 | E | S |
| *Rhodopis vesper* JOSE2 | E | S |
| *Rhodopis vesper* LSUMZB14277 | E | S |
| *Selasphorus ardens* LSUMZB52913 | C | S |
| *Selasphorus ardens* LSUMZB52914 | C | S |
| *Selasphorus ardens* LSUMZB52915 | C | S |
| *Selasphorus ardens* MBM18307 | C | S |
| *Selasphorus calliope* LSUMZB16854 | A | M |
| *Selasphorus calliope* LSUMZB23775 | A | M |
| *Selasphorus calliope* LSUMZB26272 | A | M |
| *Selasphorus calliope* MVZ170138 | A | M |
| *Selasphorus calliope* MVZ175821 | A | M |
| *Selasphorus calliope* MVZ182182 | A | M |
| *Selasphorus flammula* LSUMZB16222 | C | S |
| *Selasphorus flammula* LSUMZB19794 | C | S |
| *Selasphorus flammula* LSUMZB19847 | C | S |
| *Selasphorus flammula* LSUMZB19883 | C | S |
| *Selasphorus flammula* LSUMZB28246 | C | S |
| *Selasphorus flammula* LSUMZB28253 | C | S |
| *Selasphorus flammula* LSUMZB28260 | C | S |
| *Selasphorus flammula* LSUMZB28269 | C | S |
| *Selasphorus flammula* LSUMZB28313 | C | S |
| *Selasphorus flammula* LSUMZB9952 | C | S |
| *Selasphorus platycercus* CHIS197 | C | S |
| *Selasphorus platycercus* CHIS199 | C | S |
| *Selasphorus platycercus* CHIS200 | C | S |
| *Selasphorus platycercus* CHIS201 | C | S |
| *Selasphorus platycercus* CORO102 | A | M |
| *Selasphorus platycercus* LSUMNSB23428 | A | M |
| *Selasphorus platycercus* SWRS111 | A | M |
| *Selasphorus platycercus* SWRS112 | A | M |
| *Selasphorus platycercus* SWRS113 | A | M |
| *Selasphorus platycercus* SWRS114 | A | M |
| *Selasphorus platycercus* TLAX206 | A | S |
| *Selasphorus platycercus* TLAX207 | A | S |
| *Selasphorus rufus* LSUMZB19586 | A | M |
| *Selasphorus rufus* MVZ180196 | A | M |
| *Selasphorus rufus* SRU01HGO | A | M |
| *Selasphorus rufus* SRU02HGO | A | M |
| *Selasphorus rufus* SRU03HGO | A | M |
| *Selasphorus rufus* SRU04TLAX | A | M |
| *Selasphorus rufus* SRU05TLAX | A | M |
| *Selasphorus rufus* SRU06TLAX | A | M |
| *Selasphorus sasin* LSUMZB33988 | A | M |
| *Selasphorus sasin* LSUMZB43116 | A | M |
| *Selasphorus sasin* LSUMZB43117 | A | M |
| *Selasphorus sasin* MVZ180045 | A | M |
| *Selasphorus sasin* MVZ180632 | A | M |
| *Selasphorus sasin* MVZ182025 | A | M |
| *Selasphorus sasin* MVZ182072 | A | M |
| *Selasphorus sasin* MVZ182183 | A | M |
| *Selasphorus sasin* MVZ183552 | A | M |
| *Selasphorus scintilla* LSUMZB16266 | C | S |
| *Thaumastura cora* LSUMZB14278 | E | S |
| *Thaumastura cora* MSBBird33004 | E | S |
| *Tilmatura dupontii* KU8188 | AC | S |
| *Tilmatura dupontii* PD1 | AC | S |
| *Lamprolaima rhami* LSUMZB22001 | AC | S |
| *Eugenes fulgens* LSUMZB28291 | AC | S |
| *Panterpe insignis* LSUMZB16264 | C | S |
| *Heliomaster constantii* UWBM69186 | AC | S |
| *Heliomaster longirostris* LSUMZB18268 | ACE | S |
| *Heliomaster squamosus* FMNH392806 | E | S |
| *Heliomaster furcifer* LSUMZB6709 | E | S |
| *Lampornis amethystinus* FMHN343217 | AC | S |
| *Lampornis calolaemus* LSUMZB28169 | C | S |
| *Lampornis castaneoventris* LSUMZB28257 | C | S |
| *Lampornis calolaemus cinereicauda* LSUMZB19791 | C | S |
| *Lampornis clemenciae* LSUMZB10119 | A | S |
| *Lampornis hemileucus* LSUMZB16006 | C | S |
| *Lampornis sybillae* UWBM56159 | C | S |
| *Lampornis viridipallens* LSUMZB19268 | C | S |
| *Cynanthus sordidus* LSUMZB22004 | A | S |
| *Cynanthus sordidus* LSUMZB22005 | A | S |
| *Chlorostilbon ricordii* ANSP5570 | D | S |
| *Chlorostilbon swainsonii* AMNHNKK1017 | D | S |
| *Chlorostilbon maugaeus* LSUMZB11520 | D | S |
| *Cynanthus latirostris* LSUMZB33304 | A | S |
| *Chrolostilbon canivetii* UWBM69030 | AC | S |
| *Hylocharis leucotis* LSUMZB22003 | AC | S |
| *Campylopterus rufus* FMNH434025 | C | S |
| *Abeillia abeillei* LSUMZB22002 | AC | S |
| *Anthocephala floriceps* JVLPi1253 | E | S |
| *Amazilia cyanocephala* LSUMZB19260 | AC | S |
| *Amazilia beryllina* FMNH394217 | AC | S |
| *Amazilia rutila* UWBM56002 | AC | S |
| *Amazilia candida* UNAMPUE102 | AC | S |
