# Supplementary material for: The conquering of North America: dated phylogenetic and biogeographic inference of migratory behavior in bee hummingbirds
Source: BMC Evol Biol. 2017 Jun 5;17:126. doi: 10.1186/s12862-017-0980-5 (PMC5460336; doi:10.1186/s12862-017-0980-5)
Supplement: Supplementary file 4 — Migratory status of the Mellisugini species for ancestral state reconstruction analysis used in this study. M = migratory, S = sedentary. (DOC 57 kb) [file 12862_2017_980_MOESM4_ESM.doc]

**Additional file 4** Migratory status of the Mellisugini species for ancestral state reconstruction analysis used in this study: M = migratory, S = sedentary.

| Species | Binary  character codification | |  | Polymorphic character codification | |
| --- | --- | --- | --- | --- | --- |
|  | Scheme 1 | Scheme 2 | | | Scheme 3 |
| *Archilochus alexandri* | M | M | | | M |
| *Archilochus colubris* | M | M | | | M |
| *Atthis ellioti* | S | S | | | S |
| *Atthis heloisa* | S | S | | | S |
| *Calliphlox amethystina* | S | S | | | S |
| *Calliphlox bryantae* | S | S | | | S |
| *Calliphlox evelynae* | S | S | | | S |
| *Calliphlox lyrura* | S | S | | | S |
| *Calliphlox mitchellii* | S | S | | | S |
| *Calothorax lucifer* | M | S | | | M, S |
| *Calothorax pulcher* | S | S | | | S |
| *Calypte anna* | S | S | | | S |
| *Calypte costae* | S | S | | | S |
| *Chaetocercus bombus* | S | S | | | S |
| *Chaetocercus mulsant* | S | S | | | S |
| *Doricha eliza* | S | S | | | S |
| *Doricha enicura* | S | S | | | S |
| *Eulidia yarrellii* | S | S | | | S |
| *Mellisuga minima* | S | S | | | S |
| *Microstilbon burmeisteri* | S | S | | | S |
| *Myrmia micrura* | S | S | | | S |
| *Myrtis fanny* | S | S | | | S |
| *Rhodopis vesper* | S | S | | | S |
| *Selasphorus ardens* | S | S | | | S |
| *Selasphorus calliope* | M | M | | | M |
| *Selasphorus flammula* | S | S | | | S |
| *Selasphorus platycercus* | M | S | | | M, S |
| *Selasphorus rufus* | M | M | | | M |
| *Selasphorus sasin* | M | M | | | M |
| *Selasphorus scintilla* | S | S | | | S |
| *Thaumastura cora* | S | S | | | S |
| *Tilmatura dupontii* | S | S | | | S |
