# Supplementary material for: The conquering of North America: dated phylogenetic and biogeographic inference of migratory behavior in bee hummingbirds
Source: BMC Evol Biol. 2017 Jun 5;17:126. doi: 10.1186/s12862-017-0980-5 (PMC5460336; doi:10.1186/s12862-017-0980-5)
Supplement: Supplementary file 5 — Species names, English names and distributional range for the Mellisugini species used in this study. (DOC 75 kb) [file 12862_2017_980_MOESM5_ESM.doc]

**Additional file 5 Species names, English names and distributional range for the Mellisugini species used in this study.**

| Species | English name | Distributional range | Habitat |
| --- | --- | --- | --- |
| **CA Selasphorus** |  |  |  |
| *Selasphorus platycercus* | Broad-tailed Hummingbird | Rocky Mountains, USA; migrant to western Mexico; populations of eastern Mexico are sedentary | Interior coniferous forests |
| *Selasphorus p. guatemalae* | Broad-tailed Hummingbird | Chiapas to Guatemala | Interior coniferous forests |
| *Atthis ellioti* | Wine-throated Hummingbird | S Mexico to Honduras | Cloud forest |
| *Atthis heloisa* | Bumblebee Hummingbird | Jalisco and Tamaulipas to Oaxaca | Cloud forest |
| *Selasphorus flammula* | Volcano Hummingbird | Talamanca, CR and Chiriquí, Panama | Forest edges of mature forests |
| *Selasphorus ardens* | Glow-throated Hummingbird | Chiriquí and Veraguas, Panama | Forest edges of mature forests |
| *Selasphorus scintilla* | Scintillant Hummingbird | NC Costa Rica to W Panama | Forest edges of mature forests |
| **NA Selasphorus** |  |  |  |
| *Selasphorus calliope* | Calliope Hummingbird | Canada and USA; migrant to Mexico: Guatemala and Belize | Open montane forests and mountain meadows |
| *Selasphorus rufus* | Rufous Hummingbird | Alaska and the Pacific Northwest to California; migrant to central Mexico | Forest edges |
| *Selasphorus sasin* | Allen's Hummingbird | Coastal California from Santa Barbara north to southern Oregon | Coastal meadows |
| **Calypte** |  |  |  |
| *Calypte anna* | Anna's Hummingbird | Southern California to northern Baja California | Deserts |
| *Calypte costae* | Costa's Hummingbird | Southwestern US and Baja California Peninsula | Deserts |
| **Caribbean sheartails** |  |  |  |
| *Calliphlox evelynae* | Bahama Woodstar | Bahamas archipelago | Tropical rainforest and edges |
| *Calliphlox lyrura* | Inaguan Hummingbird | Inagua Islands | Tropical rainforest and edges |
| *Mellisuga minima* | Vervain Hummingbird | Jamaica, Hispaniola and nearby islands | Tropical rainforest and edges |
| *Mellisuga helenae* | Bee Hummibgbird | Cuba and Isla de los Pinos | Tropical rainforest and edges |
| *Archilochus colubris* | Ruby-throated Hummingbird | Eastern North America (S Canada, USA); migrant from eastern Mexico to Panama | Montane forest edges |
| *Archilochus alexandri* | Black-chinned Hummingbird | Western NA (SW Canada, USA); migrant to western Mexico | Arid |
| **Mexican sheartails** |  |  |  |
| *Doricha eliza* | Mexican Sheartail | Arid coastal slopes of central Veracruz and NW Yucatán | Arid coastal slopes and plains |
| *Doricha enicura* | Slender Sheartail | Arid slopes of Central Depression of Chiapas, Mexico to El Salvador | Arid and semideciduous tropical forests |
| *Calothorax pulcher* | Beautiful Hummingbird | Tehuacán-Cuicatlán Valley, Puebla to Central Valleys of Oaxaca | Pidemonts with xeric vegetation |
| *Calothorax lucifer* | Lucifer Hummingbird | Chihuahuan Desert from Texas to Sonora and Coahuila to arid slopes of the Sierra Madre Occidental and C Mexico | Pidemonts with xeric vegetation |
| **SA bee hummingbirds** |  |  |  |
| *Calliphlox mitchellii* | Purple-throated Woodstar | E Panama, W Colombia to W Ecuador | Mountain forests and forest edges |
| *Calliphlox bryantae* | Magenta-throated Woodstar | N Costa Rica to W Panama | Mountain forests and forest edges |
| *Chaetocercus bombus* | Little Woodstar | SW Colombia, W Ecuador to N Peru | Deciduous forest in between humid and semihumid regions |
| *Chaetocercus mulsant* | White-belllied Woodstar | C and E Colombia to C Bolivia | Humid forests edges |
| *Chaetocercus astreans* | Santa Marta Woodstar | Colombia | Mountain forests and forest edges |
| *Chaetocercus berlepschi* | Esmeraldas Woodstar | Ecuador | Edges of lowland moist forest and second growth |
| *Chaetocercus heliodor* | Gorgeted Woodstar | Colombia, Venezuela and Ecuador | Tropical mosit montane forest |
| *Microstilbon burmeisteri* | Slender-tailed Woodstar | CS Bolivia to N Argentina | Edges of humid forest |
| *Eulidia yarrellii* | Chilean Woodstar | S Peru to N Chile | Scrub and thickets along river valleys within desert regions |
| *Thaumastura cora* | Peruvian Sheartail | W Peru to N Chile | Subtropical or tropical dry shrubland |
| *Myrmia micrura* | Short-tailed Woodstar | W Ecuador to NW Peru | Subtropical or tropical dry shrubland |
| *Rhodopis vesper* | Oasis Hummingbird | NW Peru to N Chile | Desert oases, arid scrub lands and dry coastal deserts |
| *Mirtys fanny* | Purple-collared Woodstar | Ecuador to Peru | Subtropical or tropical dry shrubland |
| *Calliphlox amethystina* | Amethyst Woodstar | E Colombia to NE Argentina | Tropical moist montane forests |
| *Tilmatura dupontii* | Sparkling-tailed Woodstar | Jalisco and Veracruz to Costa Rica | Tropical moist montane forests |
