# Supplementary material for: The conquering of North America: dated phylogenetic and biogeographic inference of migratory behavior in bee hummingbirds
Source: BMC Evol Biol. 2017 Jun 5;17:126. doi: 10.1186/s12862-017-0980-5 (PMC5460336; doi:10.1186/s12862-017-0980-5)
Supplement: Supplementary file 6 — Bayesian 50% majority rule consensus trees of 132 representatives of bee hummingbirds (32 of the 36 extant species, 89%), 15 of mountain gems and 15 of emeralds. The trees are based on data sets of (a) only mitochondrial genes (‘unpartitioned mtDNA data set’), (b) only mitochondrial genes as two partitions (“partitioned mtDNA data set”), (c) only nuclear genes (‘unpartitioned nuDNA data set”), and (d) only nuclear genes as four partitions (“partitioned nuDNA data set”). Posterior probabilities (PP) > 0.5 are shown. (PDF 930 kb) [file 12862_2017_980_MOESM6_ESM.pdf]

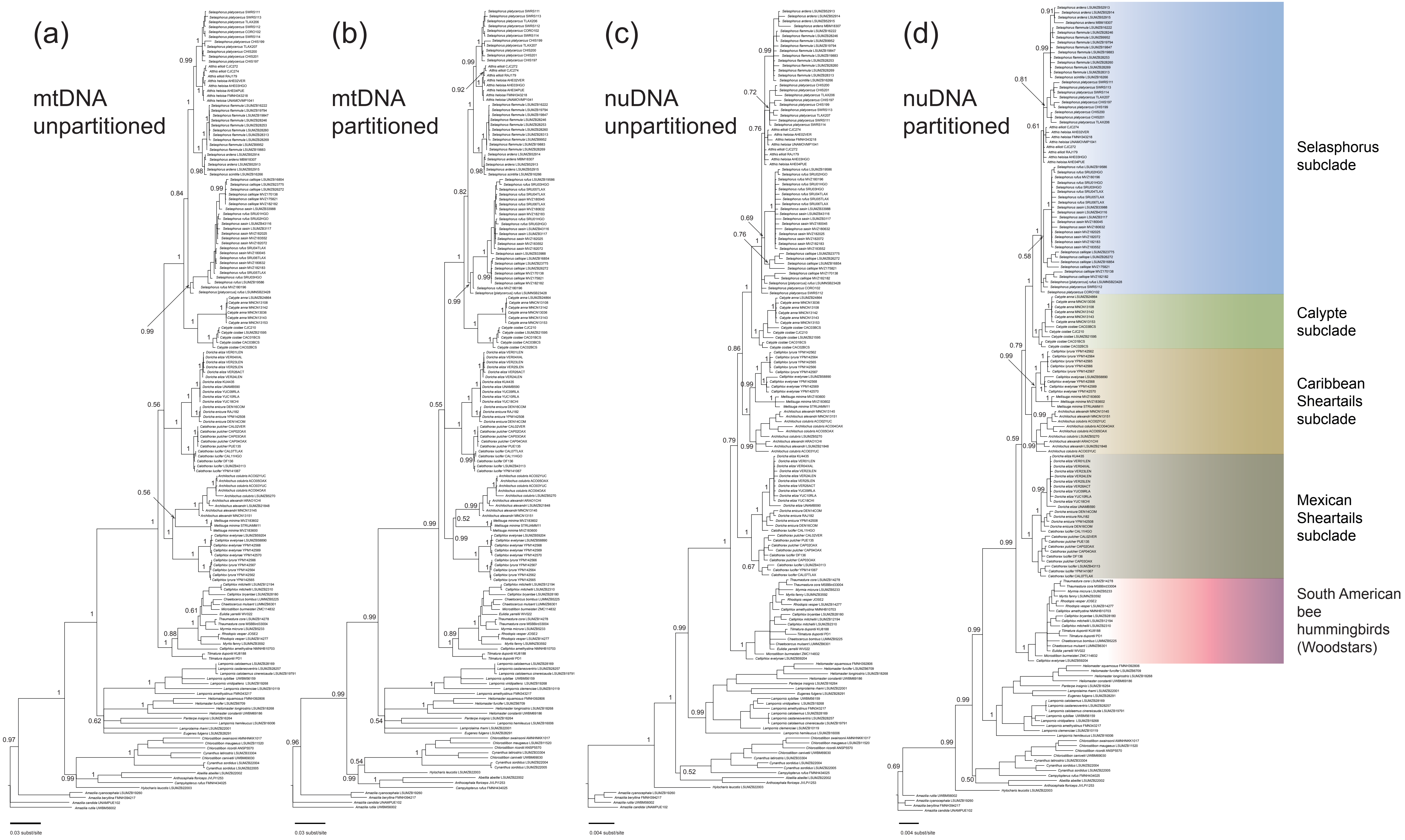

(a)
mtDNA unpartitioned
(b)
mtDNA partitioned
(c)
nuDNA unpartitioned
(d)
nuDNA partitioned
Selasphorus subclade
Calypte subclade
Caribbean Sheartails subclade
Mexican Sheartails subclade
South American bee hummingbirds (Woodstars)
0.03 subst/site
0.03 subst/site
0.004 subst/site
0.004 subst/site
