# Supplementary figures and images for: The conquering of North America: dated phylogenetic and biogeographic inference of migratory behavior in bee hummingbirds
Source: BMC Evol Biol. 2017 Jun 5;17:126. doi: 10.1186/s12862-017-0980-5 (PMC5460336; doi:10.1186/s12862-017-0980-5)

(a) McGuire *et al.* (2014)

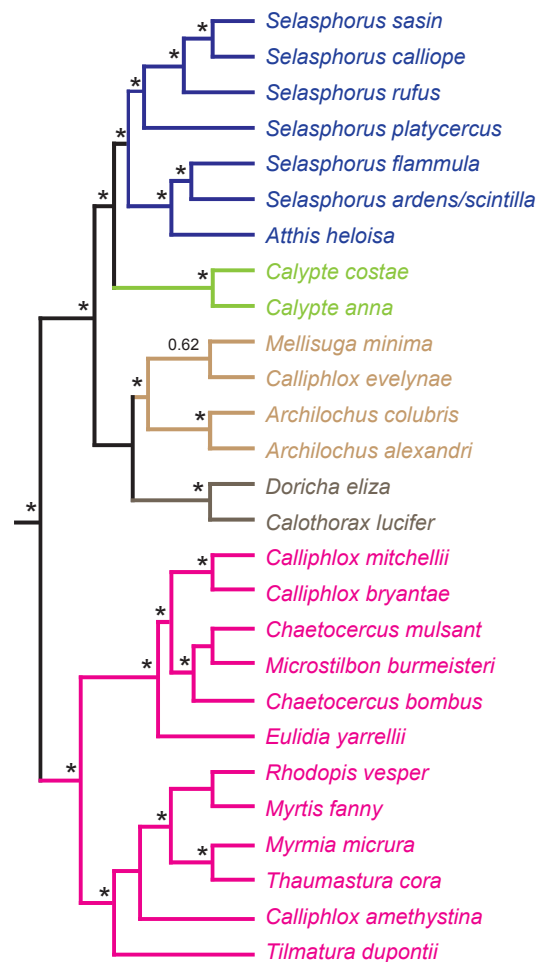

(b) Abrahamczyk & Renner (2015)

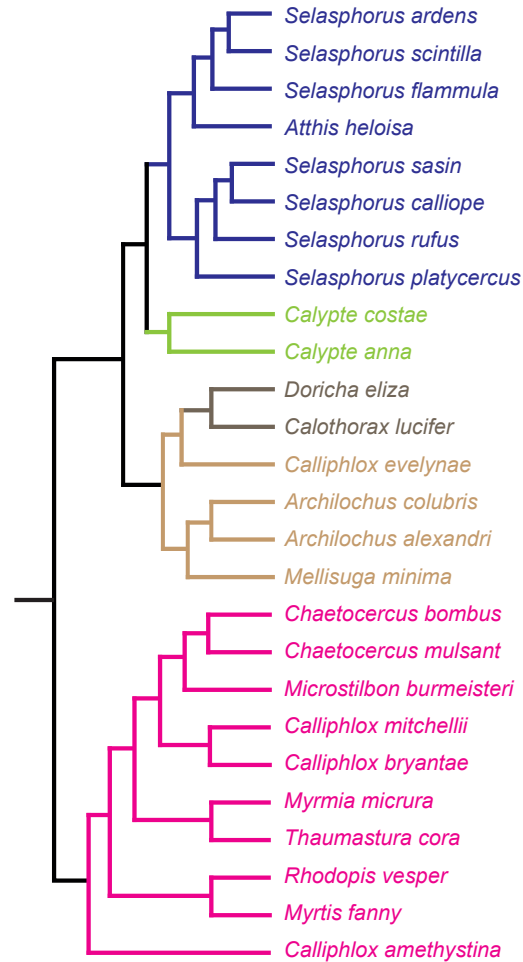

(c) This study

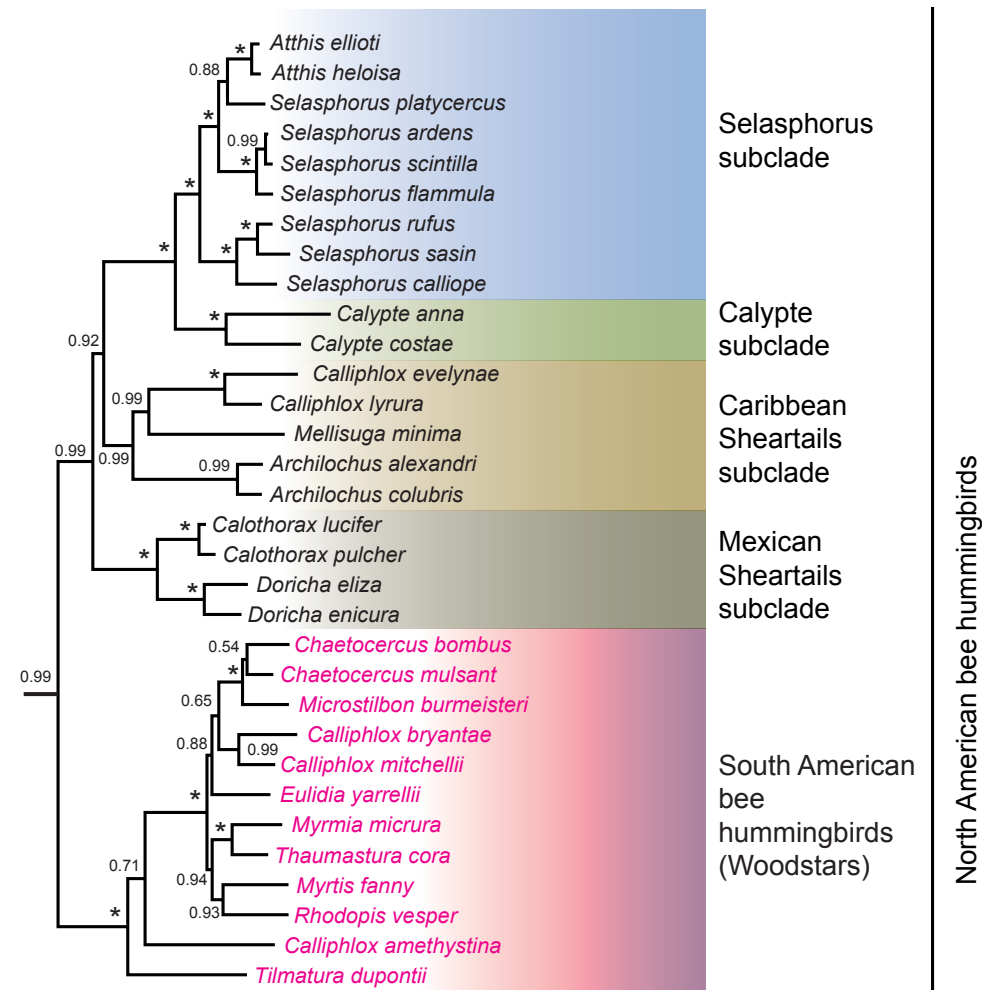

Supplement: Supplementary file 7 — Comparison of backbone tree topologies of the Mellisugini. (a) McGuire et al. [25], (b) Abrahamczyk & Renner [72], and (c) Bayesian 50% majority rule consensus tree of 32 bee hummingbird species of this study in Additional file 7. Asterisks denote nodes with 1.0 posterior probability (PP) support. Numbers at nodes reflect posterior probabilities less than 1.0. Support values for nodes of phylogeny in (b) are not provided in Abrahamczyk & Renner [72]. (PDF 425 kb) [file 12862_2017_980_MOESM7_ESM.pdf]

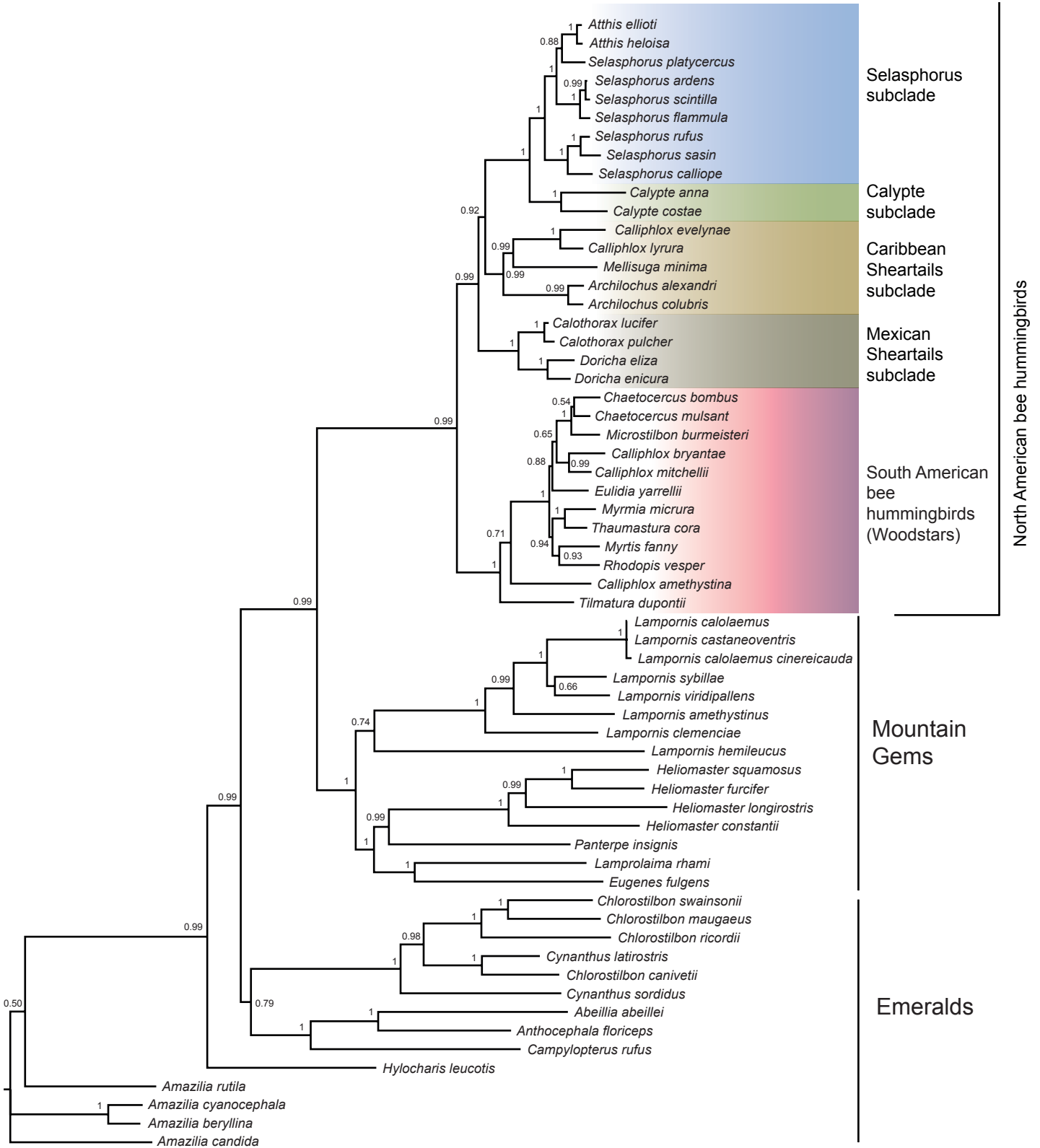

0.02 subst/site

Supplement: Supplementary file 8 — Bayesian 50% majority rule consensus tree of 32 bee hummingbird species and representatives of mountain gems and emeralds used as outgroups. The tree is based on a combined data set of all available fragments of ND2, ND4, AK1 I5, MUSK I3, ODC1 and FBG I7 and partition-specific DNA evolution models of each gene (‘6-partitions data set’). Posterior probabilities (PP) > 0.5 are shown. (PDF 404 kb) [file 12862_2017_980_MOESM8_ESM.pdf]
